# Species-Directed Therapy for Leishmaniasis in Returning Travellers: A Comprehensive Guide

**DOI:** 10.1371/journal.pntd.0002832

**Published:** 2014-05-01

**Authors:** Caspar J. Hodiamont, Piet A. Kager, Aldert Bart, Henry J. C. de Vries, Pieter P. A. M. van Thiel, Tjalling Leenstra, Peter J. de Vries, Michèle van Vugt, Martin P. Grobusch, Tom van Gool

**Affiliations:** 1 Department of Medical Microbiology, Section of Parasitology, Academic Medical Center, Amsterdam, The Netherlands; 2 Center for Tropical Medicine and Travel Medicine, Department of Infectious Diseases, Academic Medical Center, Amsterdam, The Netherlands; 3 Department of Dermatology, Academic Medical Center, Amsterdam, The Netherlands; 4 Ministry of Defence, The Hague, The Netherlands; 5 Department of Internal Medicine, Tergooi Hospitals, Hilversum, The Netherlands; New York University, United States of America

## Abstract

**Background:**

Leishmaniasis is increasingly reported among travellers. *Leishmania* species vary in sensitivity to available therapies. Fast and reliable molecular techniques have made species-directed treatment feasible. Many treatment trials have been designed poorly, thus developing evidence-based guidelines for species-directed treatment is difficult. Published guidelines on leishmaniasis in travellers do not aim to be comprehensive or do not quantify overall treatment success for available therapies. We aimed at providing comprehensive species-directed treatment guidelines.

**Methodology/Principal Findings:**

English literature was searched using PubMed. Trials and observational studies were included if all cases were parasitologically confirmed, the *Leishmania* species was known, clear clinical end-points and time points for evaluation of treatment success were defined, duration of follow-up was adequate and loss to follow-up was acceptable. The proportion of successful treatment responses was pooled using mixed effects methods to estimate the efficacy of specific therapies. Final ranking of treatment options was done by an expert panel based on pooled efficacy estimates and practical considerations. 168 studies were included, with 287 treatment arms. Based on *Leishmania* species, symptoms and geography, 25 clinical categories were defined and therapy options ranked. In 12/25 categories, proposed treatment agreed with highest efficacy data from literature. For 5/25 categories no literature was found, and in 8/25 categories treatment advise differed from literature evidence. For uncomplicated cutaneous leishmaniasis, combination of intralesional antimony with cryotherapy is advised, except for *L. guyanensis* and *L. braziliensis* infections, for which systemic treatment is preferred. Treatment of complicated (muco)cutaneous leishmaniasis differs per species. For visceral leishmaniasis, liposomal amphotericin B is treatment of choice.

**Conclusions/Significance:**

Our study highlights current knowledge about species-directed therapy of leishmaniasis in returning travellers and also demonstrates lack of evidence for treatment of several clinical categories. New data can easily be incorporated in the presented overview. Updates will be of use for clinical decision making and for defining further research.

## Author Summary

Human leishmaniasis is caused by unicellular parasites that are injected into the skin by sand-flies, small, flying insects. Many different *Leishmania* species cause various manifestations of disease, both of the skin and internal organs. Leishmaniasis is a curable disease but clear guidelines on the best available treatment are lacking. *Leishmania* species differ in sensitivity to available drugs. Until recently, identification of the infecting *Leishmania* parasite was laborious, thus therapy could not precisely be targeted to the infecting species, in contrast to many other infectious diseases. Nowadays, *Leishmania* parasites can be identified relatively easily with new DNA techniques. We studied efficacy of therapies for diseases due to different *Leishmania* species, limited to the English literature. Efficacy was summarized and presented in an easy to read format. Because of difficulties with identification of parasite species in earlier studies, quality of evidence was often limited. Our findings are a major help for clinicians to easily find optimal treatment for specific patients. Moreover, our results demonstrate where additional research is needed to further improve treatment of leishmaniasis.

## Introduction

Leishmaniasis, infection by *Leishmania* parasites, is increasingly reported among travellers, especially in adventurous and eco-tourists [1]–[6] and military personnel [7]–[11]. Three syndromes are distinguished: visceral (VL), cutaneous (CL) and mucocutaneous leishmaniasis (MCL). VL is caused by *L. donovani* and *L. infantum*, rarely by other species. If left untreated, it will generally be fatal. CL is caused by *L. major* and *L. tropica* in the Old World (OW: Europe, Africa, Asia) and by parasites of the *L. mexicana* and *L. braziliensis* complexes in Central and South America (NW, the New World). *L. infantum and L. donovani* can also cause CL. In Ethiopia and Kenya, *L. aethiopica* causes CL and diffuse cutaneous leishmaniasis, a difficult-to-treat condition. Most *L. major* and *L. mexicana* lesions heal spontaneously within 3 to 6 months [12]–[14], *L. tropica* infections within one to two years [14] but *L. braziliensis* lesions may take much longer to heal [15]. MCL, involving nose, palate and often also pharynx and larynx, is usually caused by *L. braziliensis*. *L. guyanensis*, *L. panamensis* and *L. amazonensis* rarely cause MCL. Mucosal leishmaniasis of the OW is due to extension of CL to mucosa of mouth or nose, or to local primary infection by the sand-fly; the pathophysiology is different from that of MCL. It is estimated that 500,000 new cases of VL and 1.5 million new cases of CL occur per year, resulting in loss of 2,357,000 disability-adjusted life years [16].

In non-endemic regions, experience with diagnosis and management of leishmaniasis is limited. This may lead to delay in diagnosis and an unfavourable outcome of treatment [17]. In addition to the traditional diagnostic methods of microscopy, culture and serology, molecular techniques are increasingly used. Molecular techniques allow for fast and reliable identification of the aforementioned clinically relevant *Leishmania* species [6], [18], [19].

*Leishmania* species vary in sensitivity to available drugs [20]. Current choice of treatment is mainly based on the region where the infection was acquired and on the local experience with treatment. Molecular species identification makes species-directed treatment possible [17].

Development of guidelines for the treatment of leishmaniasis remains difficult. Cochrane Reviews of the treatment of CL and MCL highlight the poverty of current information and emphasize the need for high-standard trials [21], [22]. Absence of parasitological confirmation and species characterization, lack of clearly defined treatment end-points, limited or no follow-up and small sample sizes are amongst the problems encountered, as described in the report of the expert committee of WHO [16], a report wherein the clinical responsibility of the attending health care worker is acknowledged. CL is a self-healing disease which poses particular problems for the evaluation of therapies. These problems are not addressed in many reports.

Published guidelines for the treatment of leishmaniasis in travellers do not aim to be comprehensive or do not provide an easy-to-use tool that quantifies overall success of available treatments [17], [23]–[27].

Confronted with increasing numbers of patients with leishmaniasis, we aimed at providing comprehensive, yet easily digestible treatment guidelines based on symptoms, knowledge of the *Leishmania* species involved and the region where leishmaniasis was contracted, whilst taking data quality into account.

## Methods

Literature on treatment of the leishmaniases was studied, summarized and subsequently discussed by an expert panel of the staff of the Departments of Tropical Medicine and Travel Medicine, Dermatology and Clinical Parasitology of the Academic Medical Center, Amsterdam who took into consideration patient comfort, duration of treatment, anticipated compliance with treatment, possibility of outpatient treatment, side effects and toxicity and *in vitro* data of efficacy.

### Search strategy

We searched PubMed with keywords “*Leishmania* AND therapy”, “leishmaniasis AND therapy”, “*Leishmania* AND treatment” and “leishmaniasis AND treatment” (limited to humans and published in English), from January 1979 to December 2010. Additional searches were performed on August 24^th^ 2012 and December 19^th^ 2012. All treatment studies included in Cochrane reviews on therapy for CL and MCL [21], [22], all references included in the Deutsche Gesellschaft für Tropenmedizin guidelines on therapy for VL and CL/MCL [24] and all references from reviews on treatment of leishmaniasis in travellers [2], [17], [23], [25]–[28] were considered for inclusion. Only original papers were considered.

Randomized controlled trials (RCTs) and observational studies were included according to the inclusion criteria, summarized in Table 1. If studies comprised several treatment options, separate analyses were performed for each treatment option with a minimum of 5 patients.

**Table 1:** Inclusion criteria for literature study.

Criterium number	Criterium
I.	At least 5 patients eligible for evaluation per treatment arm
II.	Parasitological proof of *Leishmania* infection by microscopy, culture or PCR
III.	Typing to species level by isoenzyme analysis, monoclonal antibodies or PCR
IV.	Well-defined clinical end-points and time points for evaluation of initial treatment with adequate duration of follow-up
V.	Loss to follow-up after initial treatment success limited to 20% in VL and MCL and 50% in CL

All criteria required for inclusion.

### Inclusion criteria of literature and considerations

#### Parasitological proof of infection

Only studies with parasitological confirmation by microscopy, culture or PCR were included with the exception of African studies of VL, that often lack parasitological confirmation due to resource-limiting circumstances. In Sudan and Ethiopia, Médecins sans Frontières (MSF) uses a diagnostic pathway based on serological diagnosis using the Direct Agglutination Test (DAT) and rK39 rapid test, both of which have been evaluated against parasitological proof [29]. Being the only studies to address VL due to *L. donovani* outside India, they were included.

#### Typing to species level

Aim of the study was to define optimal treatment based on species identification. Studies were included if the reported *Leishmania* species was typed or had been typed in earlier studies from the same area. Molecular diagnosis, isoenzyme analysis and use of monoclonal antibodies were regarded acceptable typing methods. If species identification was done for part of the isolates and more than one species had been found, the non-typed isolates were excluded, and analysis of treatment efficacy was performed on the typed isolates only. If only one species was known to cause disease in the area, both typed and non-typed isolates were included for analysis. In the Middle East where *L. major* and *L. tropica* frequently circulate together, typing was often not performed. Thus a category “*L. major/L. tropica*” was created.

#### Evaluation of treatment response

Studies with clear clinical end-points, clear time points for evaluation of initial treatment success and an adequate duration of follow-up were included. In VL, the end-points were 1) *apparent clinical cure* at the end of treatment and 2) *definite cure* without relapse after 6 months follow-up. Studies of VL in Sudan and Ethiopia often have limited follow-up. These were included as a separate category. In CL, lesions had to show initial clinical improvement as defined by the respective authors, usually evaluated after 4 to 6 weeks. Complete healing with or without scar formation had to be achieved within 3 months of treatment initiation in the case of faster-healing species (*L. major. L. mexicana*) and within 6 months in the case of slower-healing species (*L. infantum*, *L. tropica*, *L. braziliensis/L.peruviana*, *L. guyanensis*, *L. panamensis*). In MCL, complete re-epithelialisation or scarring of the lesions without signs of inflammation had to be achieved, without relapse during 12 months of follow-up.

#### Loss to follow-up

In many studies, a significant proportion of patients is lost to follow-up, especially in studies of CL in remote areas. We accepted a relatively large percentage of loss to follow-up in CL (50%) and a smaller percentage in VL and MCL (20%).

### Calculation of efficacy and pooled efficacy

To guide ranking of treatment options, results from studies were pooled to estimate the efficacy of specific therapies. For each treatment, the number of patients with a successful outcome and the total number treated with that treatment were extracted, irrespective of the dosing regimen, as only few publications report the actual dose per kg body weight [30]. If clinical end-points were related to the number of treated lesions, these were used to calculate the proportion with successful treatment success as separate analyses. Patients lost to follow-up before completing therapy were excluded from analysis. Patients who stopped therapy prematurely because of adverse events or treatment failure were regarded as treatment failures. Patients showing apparent cure that were subsequently lost to follow-up were grouped with patients classified as definite cure (best case scenario). If absolute numbers of treatment successes and failures were not reported, but the proportion of successful treatment responses and the total number of patients per study arm were known, the latter data were used to calculate the absolute numbers. For each combination of diagnosis, species, location and specific treatment, a pooled efficacy and 95% confidence interval was calculated by pooling raw proportions of successfully treated patients from individual studies using the DerSimonian-Laird random effects method after Freeman-Tukey double arcsine transformation of raw proportions using the Meta package (http://CRAN.R-project.org/package=meta). Because substantial heterogeneity between studies was expected, in particular due to differences in patient populations and study procedures, the choice was made to pool estimates using random effects, rather than fixed effects methods. Pooling was done separately for RCTs and for observational studies. Efficacy data from RCTs and observational studies were combined to arrive at the pooled efficacy of all included studies of a particular treatment.

### Presentation and ranking of treatment options

Treatments were summarized in relation to *Leishmania* species, clinical diagnosis and geography and ranked according to efficacy data from literature with type and number of studies, number of patients included and data of pooled efficacy (tables 2 and 3). Final ranking, with therapy of choice (TC) and up to 3 alternative therapies (AT 1–3), was done by the expert panel using the criteria mentioned above. Dosages for treatment options mentioned in tables 2 and 3 are provided in table 4.

**Table 2:** Species directed therapy for leishmaniasis from the “Old World.”

Cat.^a^	Species	Diagnosis	Proposed treatment	Pooled efficacy (95% CI^a^)	Randomized controlled trials			Observational studies			References
					N analyses	N patients	pooled efficacy	N analyses	N patients	pooled efficacy	
1	*L.donovani*	VL from South Asia^f^	TC^a^: liposomal amphotericin B	97% (96%–98%)^b^	48^b^	4566	98%	23^b^	2006	97%	[58], [59], [89]–[123]
			AT^a^1: miltefosine	97% (94%–99%)	7	389	99%	14	1238	94%	[106], [124]–[130]
			AT2: systemic antimony	66% (58%–74%)	16	883	67%	4	612	62%	[95], [113], [119], [122], [123], [131]–[140]
2	*L.donovani*	VL from East Africa^g^	TC: liposomal amphotericin B	83% (67%–95%)^b^	0	0	N/A	7^b^	278	83%	[89], [141], [142]
			AT1: miltefosine	79% (73%–84%)	1	221	79%	0	0	N/A	[143]
			AT2: systemic antimony	89% (85%–93%)	15	1063	89%	4	6209	89%	[61], [141], [143]–[153]
3	*L.infantum*	VL child^h^	TC: liposomal amphotericin B	98% (94%–100%)^b^	4^b^	106	97%	10^b^	156	97%	[62], [154]–[158]
			AT1: systemic antimony	94% (85%–99%)	0	0	N/A	2	62	94%	[62], [158]
4		VL adult^h^	TC: liposomal amphotericin B	100% (93%–100%)^b^	2^b^	29	100%	0	0	N/A	[155]
			AT1: systemic antimony	N/A	0	0	N/A	0	0	N/A	N/A
			AT2: miltefosine	N/A	0	0	N/A	0	0	N/A	N/A
5	*L.donovani*	CL	TC: local antimony & cryotherapy^d^	N/A	0	0	N/A	0	0	N/A	N/A
			AT1: local antimony	100% (98%–100%)	1	83	100%	0	0	N/A	[159]
			AT2: miltefosine	N/A	0	0	N/A	0	0	N/A	N/A
6	*L.infantum*	CL	TC: local antimony & cryotherapy^d^	100% (68%–100%)	0	0	N/A	1	5	100%	[157]
			AT1: miltefosine	N/A	0	0	N/A	0	0	N/A	N/A
7	*L.tropica*	CL	TC: local antimony & cryotherapy^d^	78% (69%–86%)	1	95	78%	0	0	N/A	[160]
			AT1: local antimony	76% (56%–91%)	3	327	68%	2	556	85%	[42], [47], [65], [161]–[163]
				[76% (61%–89%)]^c^	[1]	[38]^c^	[76%]^c^				
			AT2: heat therapy	69% (60%–78%)	1	108	69%	0	0	N/A	[65]
8		complex CL	TC: systemic antimony	46% (33%–59%)	3	157	39%	2	110	61%	[65], [162], [164]–[166]
			AT1: miltefosine	N/A	0	0	N/A	0	0	N/A	N/A
9	*L.major*	CL	TC: local antimony & cryotherapy^d^	83% (69%–94%)	0	0	N/A	1	36	83%	[10]
			AT1: local antimony	86% (59%–100%)	2	130	88%	2	122	82%	[10], [167]–[170]
				[73% (61%–83%]^c^	[1]^c^	[66]^c^	[73%]^c^				
			AT2: heat therapy	N/A	0	0	N/A	0	0	N/A	N/A
10		complex CL	TC: miltefosine	85% (75%–93%)	1	32	81%	1	34	88%	[66], [67]
			AT1: systemic antimony	69% (51%–84%)	5	219	59%	2	228	88%	[64], [66], [67], [167], [170]–[174]
				[68% (56%–78%)]^c^	[1]^c^	[68]^c^	[68%]^c^				
11	*L.tropica/*	CL	TC: local antimony & cryotherapy^d^	[91% (87%–94%)]^c^	[3]^c^	[264]^c^	[91%]^c^	0	0	N/A	[45], [175]
	*L.major*		AT1: local antimony	54% (41%–66%)	7	302	54%	0	0		[38], [45], [175]–[187]
				[82% (71%–91%)]^c^	[8]^c^	[1695]^c^	[80%]^c^	[1]^c^	[130]^c^	[95%]^c^	
			AT2: heat therapy	82% (73%–89%)	1	57	81%	2	39	83%	[182], [186], [188]–[190]
				[93% (89%–96%)]^c^	[2]^c^	[257]^c^	[93%]^c^				
12		complex CL	TC: systemic antimony	63% (3%–100%)	2	128	63%	0	0	N/A	[189], [191]–[193]
				[61% (13%–98%)]^c^	[2]^c^	[268]^c^	[61%]^c^				
			AT1: miltefosine	N/A	0	0	N/A	0	0	N/A	N/A
13	*L.aethiopica*	CL localized^e^	TC: local antimony & cryotherapy	N/A	0	0	N/A	0	0	N/A	N/A
			AT1: systemic antimony	90% (71%–100%)	0	0	N/A	1	19	90%	[68]

a Abbreviations used: Cat.: category, TC: treatment of choice, AT: alternative treatment, CI: confidence interval, N/A: not available, VL, CL and MCL: visceral, cutaneous and mucocutaneous leishmaniasis.

b Pooled efficacy is calculated from studies that may include Fungizone, Ambisome, Abelcet, Amphocil.

c Data between square brackets refer to studies describing number of healed lesions rather than number of healed patients.

d A wait-and-see policy may be considered.

e Diffuse cutaneous leishmaniasis requires systemic therapy.

f Efficacy data are obtained from a population with low HIV endemicity.

g Efficacy data are obtained from a population with high HIV endemicity.

h Efficacy data are obtained from immunocompetent patients only; HIV-positive patients were excluded from analysis.

**Table 3:** Species directed therapy for leishmaniasis from the “New World.”

Cat.^a^	Species	Diagnosis	Proposed treatment	Pooled efficacy (95% CI)^a^	Randomized controlled trials			Observational studies			References
					N analyses	N patients	pooled efficacy	N analyses	N patients	pooled efficacy	
14	*L.infantum*	VL^e^	TC^a^: liposomal amphotericin B	100% (93%–100%)^b^	0	0	N/A	3^b^	31	100%	[194], [195]
	(*“L.chagasi”*)		AT^a^1: systemic antimony	100% (85%–100%)	1	11	100%	0	0	N/A	[196]
			AT2: miltefosine	N/A	0	0	N/A	0	0	N/A	N/A
15	*L.infantum*	CL	TC: local antimony & cryotherapy^c^	N/A	0	0	N/A	0	0	N/A	N/A
	*(“L. chagasi”)*		AT1: miltefosine	N/A	0	0	N/A	0	0	N/A	N/A
16	*L.mexicana*	CL	TC: local antimony & cryotherapy^c^	N/A	0	0	N/A	0	0	N/A	N/A
			AT1: heat therapy	95% (91%–98%)	0	0	N/A	1	191	95%	[69]
			AT2: systemic antimony	89% (26%–100%)	1	7	57%	1	48	100%	[197], [198]
17	*L.amazonensis*	CL	TC: local antimony & cryotherapy^d^	N/A	0	0	N/A	0	0	N/A	N/A
			AT1: systemic antimony	100% (97%–100%)	0	0	N/A	1	61	100%	[199]
			AT2: liposomal amphotericin B	N/A	0	0	N/A	0	0	N/A	N/A
			AT3: miltefosine	N/A	0	0	N/A	0	0	N/A	N/A
18	*L.amazonensis*	MCL	very rare, see *L. braziliensis* MCL.	N/A	0	0	N/A	0	0	N/A	N/A
19	*L.braziliensis/*	CL	TC: systemic antimony	78% (67%–87%)	16	329	78%	6	408	77%	[77], [197], [200]–[216]
	*L.peruviana*		AT1: liposomal amphotericin B	N/A	0	0	N/A	0	0	N/A	N/A
			AT2: miltefosine	61% (24%–92%)	3	109	61%	0	0	N/A	[71], [77], [216]
20	*L.braziliensis/*	MCL	TC: systemic antimony + pentoxifylline	97% (81%–100%)	1	11	100%	1	10	90%	[81], [82]
	*L.peruviana*		AT1: systemic antimony	53% (40%–65%)	5	104	54%	2	93	50%	[78], [82], [217]–[220]
			AT2: liposomal amphotericin B	74% (40%–98%)^b^	0	0	N/A	4^b^	39	74%	[78]–[80]
			AT3: miltefosine	71% (62%–79%)	0	0	N/A	2	109	71%	[80], [221]
21	*L.panamensis*	CL	TC: local antimony & cryotherapy	N/A	0	0	N/A	0	0	N/A	N/A
			AT1: systemic antimony	75% (63%–85%)	11	291	73%	2	277	82%	[30], [216], [222]–[229]
			AT2: miltefosine	83% (61%–97%)	3	98	83%	0	0	N/A	[71], [216], [226]
			AT3: pentamidine isethionate	N/A	0	0	N/A	0	0	N/A	N/A
22	*L.panamensis*	MCL	rare, see *L. braziliensis* MCL.	N/A	0	0	N/A	0	0	N/A	N/A
23	*L.guyanensis*	CL	TC: pentamidine isethionate	87% (78%–93%)	0	0	N/A	5	745	87%	[230]–[232]
			AT1: local antimony & cryotherapy	N/A	0	0	N/A	0	0	N/A	N/A
			AT2: miltefosine	74% (61%–85%)	1	53	74%	0	0	N/A	[233]
24		MCL	very rare, see *L. braziliensis* MCL.	N/A	0	0	N/A	0	0	N/A	N/A
25	*L.naiffi*	CL	TC: local antimony & cryotherapy^c^	N/A	0	0	N/A	0	0	N/A	N/A
			AT1: pentamidine isethionate	N/A	0	0	N/A	0	0	N/A	N/A

a Abbreviations used: Cat.: category, TC: treatment of choice, AT: alternative treatment, CI: confidence interval, N/A: not available, VL, CL and MCL: visceral, cutaneous and mucocutaneous leismaniasis.

b Pooled efficacy is calculated from studies that may include Fungizone, Ambisome, Abelcet, Amphocil.

c A wait-and-see policy may be considered.

d Diffuse cutaneous leishmaniasis requires systemic therapy.

e Efficacy data are obtained from a population with low HIV endemicity.

**Table 4:** Leishmaniasis in travellers: Treatment options and dosages.†

Clinical manifestation	Patient characteristics	Dosage of treatment options
Visceral leishmaniasis	Immunocompetent patients	liposomal amphotericin B i.v.*, total dose 20 mg/kg in 2–7 days, preferably 10 mg/kg o.d.* on 2 consecutive days
		miltefosine, oral, 150 mg/d (in 2–3 doses), 28 d
		pentavalent antimony i.v. or i.m.*, 20 mg/kg o.d.*, 28 d
	Immunodeficient patients	liposomal amphotericin B i.v.*, total dose 40 mg/kg, in 4–8 d****
		miltefosine, 150 mg/d (in 2–3 doses), 6 weeks
		pentavalent antimony, as above, may have to be prolonged or repeated
	Immunodeficient patients with HIV-infection and CD 4<350/mm^3^	“secondary prophylaxis” until CD4>350/mm^3^ for at least 3 months. Type, dose, interval of secondary prophylaxis is not well established; consult expert.
Cutaneous leishmaniasis		“wait and see”
		intralesional antimony***** + cryotherapy; 3× with interval of 1 to 2 days
		miltefosine, oral, 150 mg/d (in 2–3 doses), 28 d
		pentavalent antimony, i.v. or i.m.*, 20 mg/kg/o.d., 10–20 d**
		liposomal amphotericin B, total dose 20 mg/kg, in 5 d
		pentamidine i.v., 7 mg/kg/d o.d.*, 2×, day 1 and 3***
		heat therapy
		15% paromomycine +12% methylbenzatine ointment or 15% paromomycin +0.5% gentamicin ointment (WR 279,396)
Mucocutaneous leishmaniasis, New World		pentavalent antimony, as above, + pentoxyfilline, oral 3×400 mg/d, 28 d
		liposomal amphotericin B, total dose 40 mg/kg in 4–8 d****
		miltefosine, 150 mg/d (in 2–3 doses), 28 d (may be prolonged, e.g. 42 d)
Mucosal leishmaniasis, Old World		pentavalent antimony, i.v. or i.m.*, 20 mg/kg/d o.d., 28 d
		liposomal amphotericin B, total dose 20 to 40 mg/kg in 4–8 d****
		miltefosine, 150 mg/d (in 2–3 doses), 28 d

†: details to recommendations of tables 2 and 3.

*i.v.: intravenously, i.m.: intramuscularly, o.d.: once per day.

** see text for different dosages for different species.

*** for *L.guyanensis*.

**** formal studies on a dose of 5 mg/kg are not available.

***** this treatment is technically prohibited in the US due to the absence of an IND protocol.

### Combining efficacy data of treatment with different amphotericin B formulations

Because of toxicity of amphotericin B, liposomal vehicles have been developed, notably liposomal amphotericin B (Ambisome), amphotericin B-lipid complex (Abelcet) and amphotericin B colloidal dispersion (Amphocil). All formulations are effective antileishmanial drugs, provided an adequate total dose of amphotericin B is given. Therefore, we pooled the treatment results of all amphotericin B formulations, including non-liposomal amphotericin B (deoxycholate). Liposomal formulations are recommended because higher doses can be given in short courses with limited toxicity. Ambisome is preferred as it is the most widely used and evaluated of the available formulations, it is registered in many countries and is available at reduced price in endemic countries [16] and for free for selected poor countries [16]. Moreover, costs are carried by insurance companies in several industrialized countries.

### Treatment options and patient subgroups not included in the study

Azole drugs (ketoconazole, fluconazole and itraconazole) have been applied in the treatment of cutaneous and mucocutaneous leishmaniasis [31]–[39] although their efficacy is still debated [36]. Recent papers suggest that doses might not have been adequate [40], [41]. More effective, better evaluated alternatives that require shorter treatment duration are available. We consider the azole drugs ‘reserve drugs’; they were not included in the analysis.

Studies on cryotherapy [42]–[46] and intralesional antimony treatment (ilSb^v^) [42], [45]–[47] as monotherapy have shown that these are effective treatments of *L. tropica* and *L. major* infections. Recently, ilSb^v^ has been applied in CL from the New World too [48]. Literature on cryotherapy and ilSb^v^ as monotherapy is provided, but these studies have not been included because several trials have shown superior effect of combination therapy [45], [46], [49], which we therefore prefer.

Aminosidine (paromomycin) ointment (15% aminosidine with 12% methylbenzethonium) proved effective for treatment of *L. major/tropica* and *L.mexicana* infections [50]. A new formulation of aminosidine with gentamicin, WR 279,396, shows very promising results [51], [52]. Moreover, parenteral aminosidine for VL has recently become available for endemic countries [53]. If proven effective, topical aminosidine would be a welcome treatment especially for children for whom new treatments are eagerly awaited [54]. Registration and availability of these topical formulations are limited; they are not discussed but referred to and mentioned in Table 4.

Combination therapy is advocated for endemic areas in particular, in order to prevent development of resistance. Available data are limited [53], [55]. The contribution of travellers to development of resistance is negligible and combination treatment is not considered here.

Until the introduction and wide application of HAART, HIV-*Leishmania* co-infection was a frequent problem in southern European countries [56]. More recently, this has become a major problem in eastern Africa [57]. Treatment has been diverse and no universal guideline was developed. Guidelines for secondary prophylaxis (prevention of relapses after apparent cure) were never thoroughly evaluated. Treatment reports focusing exclusively on HIV-*Leishmania* co-infection and secondary prophylaxis before HAART are not included.

## Results

One-hundred-sixty-eight clinical studies were included for final analysis, including 287 separate analyses. Data on recommended therapy and dosages are summarized in Tables 2, 3 and 4. Based on infecting *Leishmania* species, symptoms and geography, 25 clinical categories were defined: 13 in the Old World and 12 in the New World, comprising 5 and 7 *Leishmania* species, respectively. For each category, different therapy options (TC, Treatment of choice and AT 1–3, Alternative treatment options 1–3) were proposed with references added for each treatment option. Forest plots comparing proportions cured per study-arm are provided as supplementary information for each therapy option per category that was analysed.

In 12 of 25 categories treatment of choice (TC) is in agreement with highest efficacy data from the literature. In 13/25 categories, TC was not supported by this form of evidence, but depended more on expert opinion. For 5/25 categories (15, 18, 22, 24, 25) no English literature on treatment fulfilling the inclusion criteria could be identified, and for 5/25 (5, 13, 16, 17, 21) categories no literature on TC was available, although literature was available for alternative treatment (AT) in the same category. For 3/25 categories (2, 9, 11) literature evidence for TC was slightly lower than for AT in the same category (tables 2 and 3). Reasons for the preferences of the expert panel in these categories, respectively in VL in East Africa (2) and CL in the Old World (9,11) are mentioned below.

### Comments on proposed therapy

#### VL caused by *L. donovani* from the Indian subcontinent (cat. 1)

Almost all included studies were from Bihar, India, where antimony (Sb^V^) is no longer advised because of resistance (table 2). Efficacy of amphotericin B is high. Recent studies from India showed 96% and 100% cure rates after a single dose of liposomal amphotericin B of 10 mg/kg and 15 mg/kg, respectively [58], [59]. Current advise of WHO for treatment in India, Bangladesh, Bhutan or Nepal is liposomal amphotericin B, total dose 15 mg/kg in 3–5 days or a single dose of 10 mg/kg [16]. Oral treatment with miltefosine is a proven effective alternative. In India, HIV-rates are still low, thus results reflect efficacy in immunocompetent patients.

#### VL caused by *L. donovani* from East Africa (cat. 2)

Antimony resistance is infrequent in Africa; systemic (i.v.) antimony, 20 mg/kg Sb^v^, once per day (o.d.) for 28 days remains effective but comes with toxicity in HIV infected and severely malnourished patients [60], [61]. These studies include a significant number of HIV-*Leishmania* spp. co-infected patients and reflect a mixed population of immunocompetent and immunodeficient patients. For liposomal amphotericin B, WHO tends to prefer a total dose of 30 mg/kg in this region, taking into consideration that part of the patient population will be immunodeficient due to HIV infection [16]. For the immunocompetent traveller from this region, we advise liposomal amphotericin B 10 mg/kg o.d. for 2 consecutive days.

#### VL caused by *L. infantum* (including *L. chagasi*; cat. 3, 4, 14)

WHO advises liposomal amphotericin B, total dose 18–21 mg/kg in 3–6 days [16]. For children 2 doses of 10 mg/kg, o.d., on two consecutive days proved very effective [62] and is considered standard of care [25], [63]. This short regimen is likely to be effective in adults as well, but this has not been studied formally. HIV status was known for studies in the Mediterranean region and HIV-positive patients were excluded from analysis.

#### VL (caused by *L. donovani* or *L. infantum*) in the returning traveller

For the immunocompetent traveller, we propose treatment with liposomal amphotericin B, 20 mg/kg divided over 2–7 days, preferably 10 mg/kg o.d., 2 consecutive days.

#### CL of the Old World (cat. 5, 6, 7–13)

Separate categories were created for *L. tropica* and *L. major*, although recommendations for treatment are identical. Data on healed lesions (instead of healed patients) are between square brackets in Table 2.

In case of few (<5) *L. tropica* or *L. major* lesions, local therapy is preferred [16]; systemic treatment can be considered for multiple lesions, disfiguring facial lesions or lesions at sites that make topical treatment less desirable. For CL due to *L. tropica* and *L. major*, we propose combination therapy of intralesional antimony and cryotherapy, as this combination proved more effective than antimony or cryotherapy alone, although both also show high cure rates as mono-therapy [45], [46], [49]. This was based on literature and clinical experience of superior efficacy of the combination therapy by the expert panel. Heat therapy has also proven to be effective but requires special equipment [64], [65]. Miltefosine is a promising oral treatment for patients with multiple or complicated *L. major* lesions [66], [67]; evaluation of treatment of *L. tropica* infections is limited.

For CL due to *L. donovani* infection (cat. 5) or *L. infantum* (cat. 6), combined use of local antimony and cryotherapy is proposed. CL due to *L. aethiopica* (cat. 13) responds both to monotherapy with systemic antimony treatment and to monotherapy with local cryotherapy [68] suggesting efficacy of the combination of local antimony with cryotherapy.

#### CL of the New World (cat. 15–17, 19, 21, 23, 25)

The recommendations for CL due to *L. infantum* in the Old World also apply to *L. chagasi* ( = *L. infantum*) CL of the New World. For single or few lesions due to *L. mexicana* (cat. 16), local therapy (intralesional antimony with cryotherapy or heat therapy [69]) is proposed since there is no risk of MCL.

*L. mexicana* is relatively resistant to miltefosine *in vitro* [70] and *in vivo* [71], thus antimony is preferred if systemic treatment is deemed necessary.

Mucocutaneous spread due to *L. panamensis* or *L. amazonensis* is rare, suggesting that there is no need to apply systemic treatment in all cases [16]. Therefore, for single, uncomplicated lesions due to *L. panamensis* and *L. amazonensis* (cat. 17 and 21), combination therapy of local antimony and cryotherapy is advised instead of systemic therapy, although literature evidence of efficacy is lacking. Systemic treatment as for MCL due to *L. braziliensis* can be considered for complicated infections, in the absence of literature data. Systemic pentamidine is the treatment of choice for *L. guyanensis* lesions (cat. 13) in Surinam and Guyana, but recent evidence from Manaus, Brazil, shows efficacy of only about 50–60% [72]. For single, uncomplicated lesions, local therapy with antimony and cryotherapy can be considered, although MCL due to *L. guyanensis* is not as rare as formerly thought [73]. Long follow-up is advisable, as for several other leishmaniasis manifestations [74], [75]. Based on the close taxonomic relationship of *L. panamensis* and *L. guyanensis*, one would expect pentamidine to be effective for *L. panamensis* as well, but no data are available.

A case series on CL due to *L. naiffi* (cat. 25), not fulfilling the inclusion criteria of this study, suggests that local antimony and cryotherapy are effective [76].

Local therapy is not recommended for CL due to *L. braziliensis* (cat. 19), because of the risk of MCL. However, local therapy has been studied and the dogma that *L. braziliensis* infection has to be treated systemically has been challenged [16], [22]. Most reports are on systemic antimony, which is considered the gold standard. The few studies on treatment with miltefosine show comparable results, although response varies depending on geography, possibly related to differences in parasite strains [77]. We include amphotericin B formulations as alternative treatment because efficacy with this drug for treatment of MCL was at least equivalent to antimony treatment [78]–[80].

#### MCL (cat. 18, 20, 22, 24)

Included MCL treatment studies are limited to infection with *L. braziliensis* (cat. 20). Traditionally, systemic antimony was used but low success rates prompted research for alternatives. Two small studies on the combination of antimony and pentoxifylline showed high cure rates [81], [82]. We recommend this combination as therapy of choice for MCL while awaiting further information on liposomal amphotericin B and miltefosine, both of which have proven to be alternatives in small studies [78], [80].

MCL due to infection with *L. panamensis*, *L.amazonensis* and *L. guyanensis* (cat. 18, 22 and 24) is rare and literature evidence on efficacy of treatment is not available. Proposed therapy is as for MCL due to *L. braziliensis*.

## Discussion

To optimize efficacy of treatment of leishmaniasis, the clinical diagnosis of the patient, the *Leishmania* species involved, and geography of infection should be considered. With these aspects as major determinants and with results of literature studies, we created a comprehensive guideline for species-directed treatment of leishmaniasis in the returning traveller. All English literature about treatment of leishmaniasis with a minimum of five persons per treatment was analysed. Efficacy data were pooled, resulting in an overall efficacy figure. Data are presented in easy-to-read overviews (tables 2 and 3). To our knowledge, this form of analysis and presentation has not been used before in overviews for treatment of leishmaniasis. It provides fast insight in current knowledge of treatment choices in clinical practice but also highlights the missing data needed to optimize treatment of leishmaniasis.

It is difficult to define and rank evidence based treatment recommendations for leishmaniasis. Insufficiencies of reported studies include lack of parasitological proof, of species identification, clear definitions and end-points and insufficient follow-up or considerable loss to follow-up. Only few well-designed placebo-controlled trials have been performed and not all specific treatments were compared side-by-side, precluding a comprehensive meta-analysis of RCTs. Cochrane reviews revealed these limitations and called for initiatives to improve studies [21], [22], a plea supported by WHO [16].

In this study, a significant proportion of the included analyses were from observational studies (106/287 = 37%), including case-series of a single treatment regimen. In order to present a comprehensive overview of all available treatment options, we included both randomized controlled trials and observational studies. To allow estimation of pooled efficacy for all treatment modalities and across study designs, we chose a pragmatic approach of calculating the treatment success per treatment arm. As no comparisons between treated and untreated control groups were made, the external validity of the presented (pooled) efficacy estimates depends on the assumption that self-cure was negligible within the study period. However, because of similar timing of outcome assessment across studies, one may assume that when therapies for a specific species and clinical modality are compared, the degree of over-estimation of the treatment effect will have been comparable across studies, thus preserving the relative ranking of treatments which form the basis for our recommendations. A potential risk of not randomising treatment, as in observational studies, is that patients receiving a new treatment may have better or worse prognosis than the average patient. Nevertheless, pooled results from observational studies were comparable with those from RCTs (tables 2 and 3). Diverging results should be viewed with caution.

For several clinical categories as defined in this study, limited literature was available, thus capitalizing on an expert panel was important. Efficacy figures of the literature study were combined with aspects of toxicity, convenience, and possibility of out-patient treatment to develop the guideline (tables 2 and 3).

Combination of intralesional antimony with cryotherapy is advised for all cases of CL with less than 5 lesions. Exceptions are infections with *L. guyanensis* (pentamidine) and *L. braziliensis* from Bolivia, Peru and Ecuador (systemic treatment). Treatment of complicated CL and MCL differs per species. For VL in immunocompetent travellers, treatment of choice is liposomal amphotericin B, total dose 20 mg/kg in 2–7 days, preferably 10 mg/kg o.d. on 2 consecutive days. Western European travellers mostly acquire VL in the Mediterranean region, occasionally in Latin America and rarely in the Indian continent or East Africa. For immunodeficient patients a total dose of 40 mg/kg is advised, administered over 4 to 8 days.

In India, cost of treatment is a major consideration and slightly lower cure rates with lower dosages and retreatment of relapsed cases are accepted, if cost-effective. For travellers, highest cure rates and convenience are priorities and cost of drugs is less important; a reason to propose use of higher dosages of liposomal amphotericin B than are currently used in India.

*Leishmania* species differ in *in vitro* or *in vivo* sensitivity to available drugs, in risk of development of complicated disease and in time required for spontaneous healing, thus knowledge of the *Leishmania* species and/or strain involved is important. Nowadays, PCR is an established diagnostic method, and species identification by molecular methods, e.g. sequence analysis, is available at reasonable cost. With the advent of these techniques, fast, precise and relatively cheap species differentiation has come within reach of many laboratories [6], [18], [19].

There are several limitations to our study. Firstly, we restricted our literature search in PubMed to the English language and may have missed relevant studies, since there is a fairly large body of literature published in other languages, especially in Latin America.

Secondly, using the “best case scenario” for evaluation of treatment success for CL studies with considerable loss to follow-up may have led to overestimation of treatment results. However, this choice seems not unreasonable in view of the spontaneous healing tendency of at least 50% and the assumption that cured patients are less inclined to return for evaluation. Moreover, very few patients with initial cure ultimately fail treatment.

Thirdly, azole drugs and aminosidine ointments are not fully discussed for the aforementioned reasons. New developments regarding aminosidine ointment WR 279,396 are promising, in particular for treatment of children [60].

Fourthly, methods of species identification have not been standardized and the exact status of several species is debated [83]. E.g. it has been argued that *L. panamensis* is a geographically confined subcluster or subspecies of *L. guyanensis* rather than a distinct species [84], [85]. *L. guyanensis* may consist of several (sub)species or of different strains with different behaviour and different sensitivity to drugs [72]. Moreover, in many studies, species identification was not universally performed but based on prior surveys and studies, with inherent uncertainties of older, different ways of typing and possibilities of shifts of endemicity. As molecular techniques are becoming more widely available, we will likely get more and better information in the near future.

Fifthly, due to the relative scarcity of reports of non-*L. braziliensis* MCL, it is difficult to evaluate the risk of developing MCL due to *L. panamensis* or *L. amazonensis*. The decision on local or systemic treatment for CL due to these species requires an individual risk benefit analysis.

Finally, for several drugs a standard dosing schedule was reported in the included studies, but the exact doses in mg/kg for the individual patients (or a mean or median with ranges for the studied population) were hardly ever reported. Since the publication of Herwaldt and Berman [86], antimony treatment of VL is with 20 mg Sb^v^/kg o.d. during 28–30 days, and this is how antimony treatment of VL has been reported in the literature since then. Dose and duration of antimony have varied in several studies of CL of the New World but WHO [16] advises 20 mg/kg o.d., for 20 days. However, it is impossible to know if in the real world patients actually received 20 mg/kg. Are they actually weighed, and if so, by a calibrated scale? Three antimony preparations are available: meglumine antimoniate (Glucantime) containing 81 mg Sb^v^ per ml according to the WHO [16], although others [30] mention 85 mg/ml, sodium stibogluconate (Pentostam) and generic sodium stibogluconate (SSG, produced in India) both containing 100 mg Sb^v^ per ml. Glucantime is used in Latin America and French speaking countries and comes in ampoules of 5 ml [16]. There will be a tendency to use full ampoules. Pentostam is used in English speaking countries while SSG is mostly used by MSF. The latter two come in bottles of 100 ml; in general full millilitres will be used. Doses will be rounded off, upwards and downwards, regularly leading to under- or overdosing [30]. Actual total doses given per patient are rarely recalculated and reported.

This is relevant for other drugs as well, e.g. for miltefosine that comes in capsules of 50 and 100 mg. An adult with bodyweight ≥50 kg receives 3×50 mg/d. In one study on *L. major* infections this led to doses of 1.3 to 2.1 mg/kg/d [67].

The difference in individual doses is important for several drugs; too much may lead to toxicity and adverse events, too low doses to treatment failure and development of resistance. We chose a practical approach of extracting all successfully treated patients irrespective of the dosing regimen.

Our study highlights current knowledge of species-directed therapy of leishmaniasis in returning travellers. It also clearly demonstrates the lack of evidence in the literature for treatment of several clinical categories with different *Leishmania* species. More, well-designed and properly executed trials are needed to optimize advice on treatment [87]. This paucity of knowledge [21], [22] has been recognized and prompted new initiatives to improve study design, diagnosis and evaluation of studies of the leishmaniases [16], [87]. Moreover, in Europe, a study group has been established to integrate research on optimal treatment of VL, CL and MCL among travellers [88].

Updated versions of our overview will be of use both for clinical decision making and for defining further research.

## Supporting Information

Checklist S1 PRISMA checklist.
(DOC)

Diagram S1 PRISMA flow chart.
(DOC)

Figure S1 Forest plots comparing proportions leishmaniasis patients cured within treatment-arm Study: Study from which a treatment-arm on was included for analysis. Studies are divided in observational studies and randomized controlled trials (RCTs). Events: The number of patients cured in this treatment-arm. Total: The number of patients treated in this treatment-arm. Proportion: The proportion of patients that were cured in this treatment-arm. 95%-CI: 95%-confidence interval of the proportion cured. W(fixed): weight of the study-arm included in the fixed effect model. W(random): weight of the study-arm included in the random effects model.
(PDF)
