# Supplementary material for: Species-Directed Therapy for Leishmaniasis in Returning Travellers: A Comprehensive Guide
Source: PLoS Negl Trop Dis. 2014 May 1;8(5):e2832. doi: 10.1371/journal.pntd.0002832 (PMC4006727; doi:10.1371/journal.pntd.0002832)
Supplement: Diagram S1 — PRISMA flow chart. (DOC) [file pntd.0002832.s002.doc]

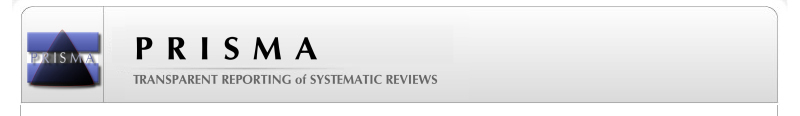
**PRISMA 2009 Flow Diagram**

**Screening**

**Included**

**Eligibility**

**Identification**

Records identified through database searching
(n = 4338)

Records identified from references 2, 17, 21 - 28
(n = 1187)

Records screened

(including duplicates)
(n = 5525)

Records and duplicates excluded
(n = 5128)

Full-text articles assessed for eligibility
(n = 397)

Full-text articles excluded
not meeting the topics and/or inclusion criteria (n= 229)

Studies included in analysis
(n = 168)
