# Supplementary material for: Species-Directed Therapy for Leishmaniasis in Returning Travellers: A Comprehensive Guide
Source: PLoS Negl Trop Dis. 2014 May 1;8(5):e2832. doi: 10.1371/journal.pntd.0002832 (PMC4006727; doi:10.1371/journal.pntd.0002832)
Supplement: Figure S1 — Forest plots comparing proportions leishmaniasis patients cured within treatment-arm Study: Study from which a treatment-arm on was included for analysis. Studies are divided in observational studies and randomized controlled trials (RCTs). Events: The number of patients cured in this treatment-arm. Total: The number of patients treated in this treatment-arm. Proportion: The proportion of patients that were cured in this treatment-arm. 95%-CI: 95%-confidence interval of the proportion cured. W(fixed): weight of the study-arm included in the fixed effect model. W(random): weight of the study-arm included in the random effects model. (PDF) [file pntd.0002832.s003.pdf]

Figure S1 - Forest plots comparing proportions cured within study-arms for treatment of patients with leishmaniasis

1a. Forest plot comparing proportions cured within study-arm for treatment with amphotericin B formulations of patients with visceral leishmaniasis caused by *L. donovani* from South Asia – observational studies

**Study**	**Events**	**Total**	**Proportion**	**95%-CI**	**W(fixed)**	**W(random)**
**study_type = Observational**						
Mishra et al. 1991	14	15	0.93	[0.68; 1.00]	0.2%	0.9%
Thakur et al. 1993	48	50	0.96	[0.86; 1.00]	0.8%	1.5%
Thakur et al. 1994	60	60	1.00	[0.94; 1.00]	0.9%	1.6%
Thakur et al. 1994	60	60	1.00	[0.94; 1.00]	0.9%	1.6%
Jha et al. 1995	31	32	0.97	[0.84; 1.00]	0.5%	1.3%
Sundar et al. 1996	21	21	1.00	[0.84; 1.00]	0.3%	1.0%
Berman et al. 1998	11	15	0.73	[0.45; 0.92]	0.2%	0.9%
Berman et al. 1998	19	20	0.95	[0.75; 1.00]	0.3%	1.0%
Berman et al. 1998	20	20	1.00	[0.83; 1.00]	0.3%	1.0%
Bodhe et al. 1999	8	11	0.73	[0.39; 0.94]	0.2%	0.7%
Bodhe et al. 1999	11	13	0.85	[0.55; 0.98]	0.2%	0.8%
Bodhe et al. 1999	11	11	1.00	[0.72; 1.00]	0.2%	0.7%
Bodhe et al. 1999	15	17	0.88	[0.64; 0.99]	0.3%	0.9%
Bodhe et al. 1999	9	10	0.90	[0.55; 1.00]	0.2%	0.7%
Thakur et al. 1999	931	938	0.99	[0.98; 1.00]	14.2%	2.3%
Sundar et al. 2000	65	70	0.93	[0.84; 0.98]	1.1%	1.7%
Sundar et al. 2003	183	200	0.92	[0.87; 0.95]	3.0%	2.1%
Thakur et al. 2004	178	178	1.00	[0.98; 1.00]	2.7%	2.1%
Sundar et al. 2009	14	15	0.93	[0.68; 1.00]	0.2%	0.9%
Sundar et al. 2009	11	15	0.73	[0.45; 0.92]	0.2%	0.9%
Sundar et al. 2009	12	15	0.80	[0.52; 0.96]	0.2%	0.9%
Sundar et al. 2009	15	15	1.00	[0.78; 1.00]	0.2%	0.9%
Sinha et al. 2010	204	205	1.00	[0.97; 1.00]	3.1%	2.1%
**Fixed effect model**		**2006**	**1.00**	**[0.99; 1.00]**	**30.5%**	**--**
**Random effects model**			**0.97**	**[0.93; 0.99]**	**--**	**28.2%**

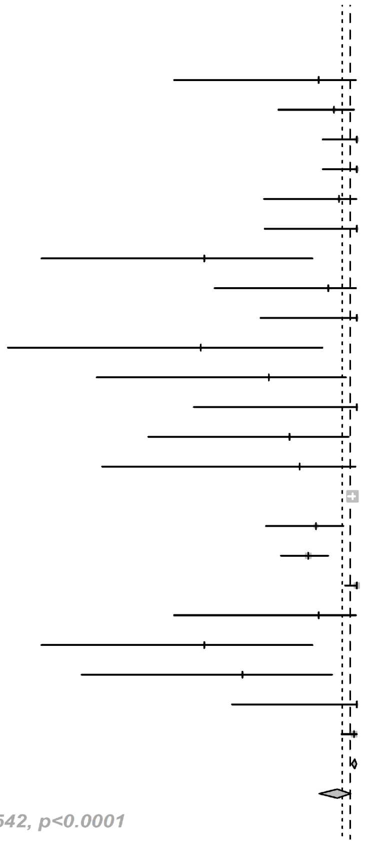

Heterogeneity: I-squared=78.9%, tau-squared=0.0542, p<0.0001

1b. Forest plot comparing proportions cured within study-arm for treatment with amphotericin B formulations of patients with visceral leishmaniasis caused by *L. donovani* from South Asia – randomized controlled trials

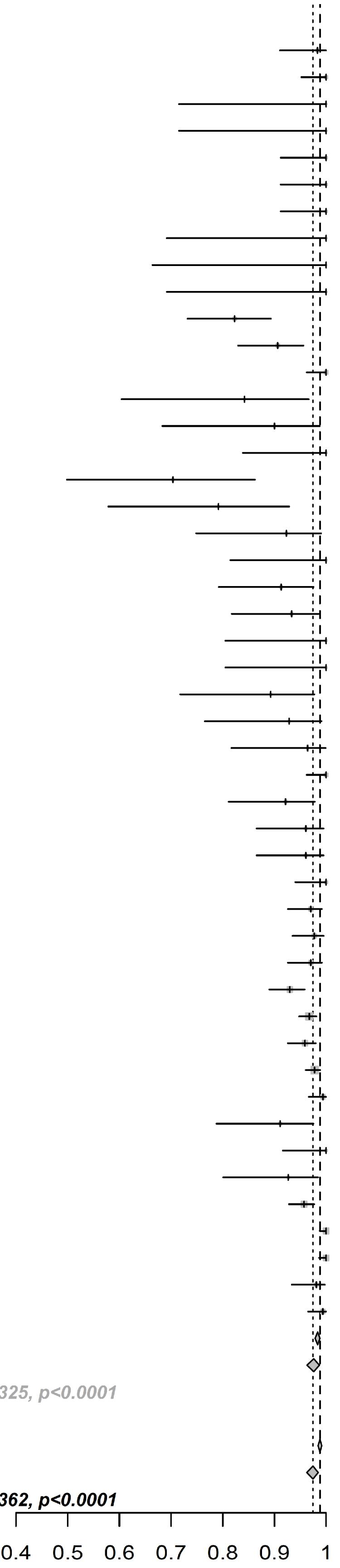

**study_type = RCT**					
Mishra et al. 1992	59	60	0.98 [0.91; 1.00]	0.9%	1.6%
Thakur et al. 1993	75	75	1.00 [0.95; 1.00]	1.1%	1.7%
Thakur et al. 1994	11	11	1.00 [0.72; 1.00]	0.2%	0.7%
Thakur et al. 1994	11	11	1.00 [0.72; 1.00]	0.2%	0.7%
Mishra et al. 1994	40	40	1.00 [0.91; 1.00]	0.6%	1.4%
Thakur et al. 1994	40	40	1.00 [0.91; 1.00]	0.6%	1.4%
Thakur et al. 1994	40	40	1.00 [0.91; 1.00]	0.6%	1.4%
Thakur et al. 1996	10	10	1.00 [0.69; 1.00]	0.2%	0.7%
Thakur et al. 1996	9	9	1.00 [0.66; 1.00]	0.1%	0.6%
Thakur et al. 1996	10	10	1.00 [0.69; 1.00]	0.2%	0.7%
Thakur et al. 1996	79	96	0.82 [0.73; 0.89]	1.5%	1.8%
Thakur et al. 1996	87	96	0.91 [0.83; 0.96]	1.5%	1.8%
Thakur et al. 1996	96	96	1.00 [0.96; 1.00]	1.5%	1.8%
Sundar et al. 1997	16	19	0.84 [0.60; 0.97]	0.3%	1.0%
Sundar et al. 1997	18	20	0.90 [0.68; 0.99]	0.3%	1.0%
Sundar et al. 1997	21	21	1.00 [0.84; 1.00]	0.3%	1.0%
Sundar et al. 1998	19	27	0.70 [0.50; 0.86]	0.4%	1.2%
Sundar et al. 1998	19	24	0.79 [0.58; 0.93]	0.4%	1.1%
Sundar et al. 1998	24	26	0.92 [0.75; 0.99]	0.4%	1.2%
Sundar et al. 1998	18	18	1.00 [0.81; 1.00]	0.3%	1.0%
Sundar et al. 2001	42	46	0.91 [0.79; 0.98]	0.7%	1.5%
Sundar et al. 2001	42	45	0.93 [0.82; 0.99]	0.7%	1.5%
Thakur et al. 2001	17	17	1.00 [0.80; 1.00]	0.3%	0.9%
Thakur et al. 2001	17	17	1.00 [0.80; 1.00]	0.3%	0.9%
Sundar et al. 2002	25	28	0.89 [0.72; 0.98]	0.4%	1.2%
Sundar et al. 2002	26	28	0.93 [0.76; 0.99]	0.4%	1.2%
Sundar et al. 2002	27	28	0.96 [0.82; 1.00]	0.4%	1.2%
Sundar et al. 2002	96	96	1.00 [0.96; 1.00]	1.5%	1.8%
Sundar et al. 2004	47	51	0.92 [0.81; 0.98]	0.8%	1.5%
Sundar et al. 2004	49	51	0.96 [0.87; 1.00]	0.8%	1.5%
Sundar et al. 2004	49	51	0.96 [0.87; 1.00]	0.8%	1.5%
Thakur et al. 2004	60	60	1.00 [0.94; 1.00]	0.9%	1.6%
Sundar et al. 2006	131	135	0.97 [0.93; 0.99]	2.1%	2.0%
Sundar et al. 2006	129	132	0.98 [0.94; 1.00]	2.0%	2.0%
Sundar et al. 2006	131	135	0.97 [0.93; 0.99]	2.1%	2.0%
Sundar et al. 2007	225	242	0.93 [0.89; 0.96]	3.7%	2.1%
Sundar et al. 2007	476	492	0.97 [0.95; 0.98]	7.5%	2.2%
Sundar et al. 2007	234	244	0.96 [0.93; 0.98]	3.7%	2.1%
Sundar et al. 2007	483	494	0.98 [0.96; 0.99]	7.5%	2.2%
Sundar et al. 2007	163	164	0.99 [0.97; 1.00]	2.5%	2.0%
Sundar et al. 2008	41	45	0.91 [0.79; 0.98]	0.7%	1.5%
Thakur et al. 2008	42	42	1.00 [0.92; 1.00]	0.6%	1.4%
Das et al. 2009	38	41	0.93 [0.80; 0.98]	0.6%	1.4%
Sund et al. 2010	291	304	0.96 [0.93; 0.98]	4.6%	2.2%
Singh et al. 2010	299	299	1.00 [0.99; 1.00]	4.5%	2.2%
Singh et al. 2010	267	267	1.00 [0.99; 1.00]	4.0%	2.1%
Sundar et al. 2010	104	106	0.98 [0.93; 1.00]	1.6%	1.9%
Sundar et al. 2011	156	157	0.99 [0.97; 1.00]	2.4%	2.0%
**Fixed effect model**		**4566**	**0.98 [0.98; 0.99]**	**69.5%**	**--**
**Random effects model**			**0.98 [0.96; 0.99]**	**--**	**71.8%**
Heterogeneity: I-squared=75.1%, tau-squared=0.0325, p<0.0001					
**Fixed effect model**		**6572**	**0.99 [0.98; 0.99]**	**100%**	**--**
**Random effects model**			**0.97 [0.96; 0.98]**	**--**	**100%**
Heterogeneity: I-squared=76.5%, tau-squared=0.0362, p<0.0001					

2. Forest plot comparing proportions cured within study-arm for treatment with miltefosine of patients with visceral leishmaniasis caused by *L. donovani* from South Asia

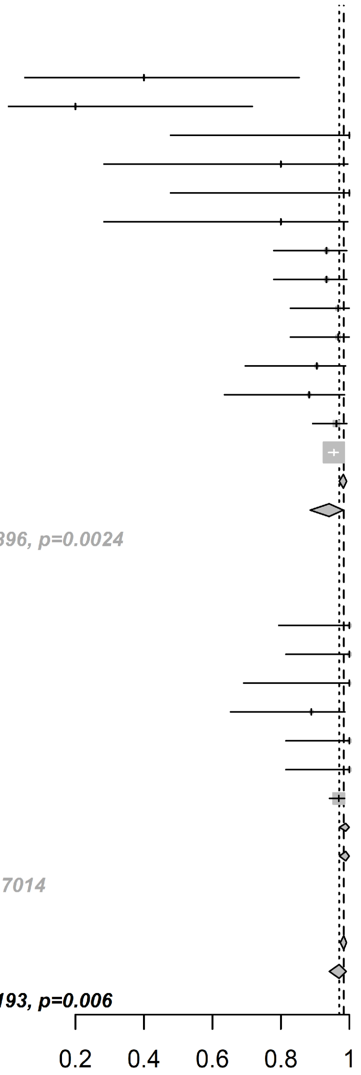

Study	Events	Total	Proportion	95%-CI	W(fixed)	W(random)
study_type = Observational						
Sundar et al. 1998	2	5	0.40	[0.05; 0.85]	0.3%	1.5%
Sundar et al. 1998	1	5	0.20	[0.01; 0.72]	0.3%	1.5%
Sundar et al. 1998	5	5	1.00	[0.48; 1.00]	0.3%	1.5%
Sundar et al. 1998	4	5	0.80	[0.28; 0.99]	0.3%	1.5%
Sundar et al. 1998	5	5	1.00	[0.48; 1.00]	0.3%	1.5%
Sundar et al. 1998	4	5	0.80	[0.28; 0.99]	0.3%	1.5%
Jha et al. 1999	28	30	0.93	[0.78; 0.99]	1.9%	5.7%
Jha et al. 1999	28	30	0.93	[0.78; 0.99]	1.9%	5.7%
Jha et al. 1999	29	30	0.97	[0.83; 1.00]	1.9%	5.7%
Jha et al. 1999	29	30	0.97	[0.83; 1.00]	1.9%	5.7%
Sundar et al. 2003	19	21	0.90	[0.70; 0.99]	1.3%	4.5%
Sundar et al. 2003	15	17	0.88	[0.64; 0.99]	1.1%	3.9%
Bhattacharya et al. 2004	76	79	0.96	[0.89; 0.99]	4.9%	9.4%
Bhattacharya et al. 2007	927	971	0.95	[0.94; 0.97]	59.3%	14.7%
Fixed effect model		1238	0.98	[0.97; 0.99]	76.0%	--
Random effects model			0.94	[0.89; 0.98]	--	64.3%
Heterogeneity: I-squared=59.3%, tau-squared=0.0396, p=0.0024						
study_type = RCT						
Sundar et al. 1999	16	16	1.00	[0.79; 1.00]	1.0%	3.7%
Sundar et al. 1999	18	18	1.00	[0.81; 1.00]	1.1%	4.1%
Sundar et al. 1999	10	10	1.00	[0.69; 1.00]	0.6%	2.6%
Sundar et al. 2000	16	18	0.89	[0.65; 0.99]	1.1%	4.1%
Sundar et al. 2000	18	18	1.00	[0.81; 1.00]	1.1%	4.1%
Sundar et al. 2000	18	18	1.00	[0.81; 1.00]	1.1%	4.1%
Sundar et al. 2002	282	291	0.97	[0.94; 0.99]	17.8%	13.1%
Fixed effect model		389	0.99	[0.97; 1.00]	24.0%	--
Random effects model			0.99	[0.97; 1.00]	--	35.7%
Heterogeneity: I-squared=0%, tau-squared=0, p=0.7014						
**Fixed effect model**		**1627**	**0.98**	**[0.97; 0.99]**	**100%**	**--**
**Random effects model**			**0.97**	**[0.94; 0.99]**	**--**	**100%**
Heterogeneity: I-squared=49.2%, tau-squared=0.0193, p=0.006						

3. Forest plot comparing proportions cured within study-arm for treatment with systemic antimony of patients with visceral leishmaniasis caused by *L. donovani* from South Asia

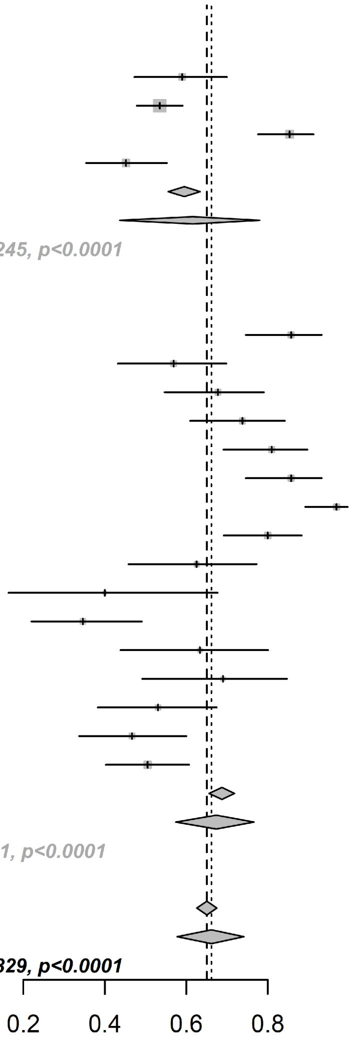

Study	Events	Total	Proportion	95%-CI	W(fixed)	W(random)
study_type = Observational						
Thakur et al. 1998	46	78	0.59	[0.47; 0.70]	5.2%	5.2%
Sundar et al. 2000	168	314	0.54	[0.48; 0.59]	20.9%	5.6%
Rijal et al. 2003	99	116	0.85	[0.78; 0.91]	7.7%	5.3%
Thakur et al. 2004	47	104	0.45	[0.35; 0.55]	6.9%	5.3%
Fixed effect model		612	0.60	[0.56; 0.63]	40.8%	--
Random effects model			0.62	[0.44; 0.78]	--	21.4%
Heterogeneity: I-squared=94.4%, tau-squared=0.1245, p<0.0001						
study_type = RCT						
Thakur et al. 1984	54	63	0.86	[0.75; 0.93]	4.2%	5.1%
Thakur et al. 1988	33	58	0.57	[0.43; 0.70]	3.9%	5.0%
Thakur et al. 1988	42	62	0.68	[0.55; 0.79]	4.2%	5.1%
Thakur et al. 1988	45	61	0.74	[0.61; 0.84]	4.1%	5.1%
Thakur et al. 1988	51	63	0.81	[0.69; 0.90]	4.2%	5.1%
Thakur et al. 1988	54	63	0.86	[0.75; 0.93]	4.2%	5.1%
Thakur et al. 1988	62	64	0.97	[0.89; 1.00]	4.3%	5.1%
Thakur et al. 1993	60	75	0.80	[0.69; 0.88]	5.0%	5.2%
Mishra et al. 1994	25	40	0.62	[0.46; 0.77]	2.7%	4.8%
Sundar et al. 1995	6	15	0.40	[0.16; 0.68]	1.0%	3.8%
Sundar et al. 1997	18	52	0.35	[0.22; 0.49]	3.5%	5.0%
Jha et al. 1998	19	30	0.63	[0.44; 0.80]	2.0%	4.6%
Thakur et al. 2000	20	29	0.69	[0.49; 0.85]	2.0%	4.5%
Thakur et al. 2000	26	49	0.53	[0.38; 0.67]	3.3%	4.9%
Thakur et al. 2004	28	60	0.47	[0.34; 0.60]	4.0%	5.1%
Thakur et al. 2008	50	99	0.51	[0.40; 0.61]	6.6%	5.3%
Fixed effect model		883	0.69	[0.66; 0.72]	59.2%	--
Random effects model			0.67	[0.57; 0.77]	--	78.6%
Heterogeneity: I-squared=89%, tau-squared=0.1471, p<0.0001						
**Fixed effect model**		**1495**	**0.65**	**[0.63; 0.67]**	**100%**	**--**
**Random effects model**			**0.66**	**[0.58; 0.74]**	**--**	**100%**
Heterogeneity: I-squared=90.6%, tau-squared=0.1329, p<0.0001						

4. Forest plot comparing proportions cured within study-arm for treatment with amphotericin B formulations of patients with visceral leishmaniasis caused by *L. donovani* from East Africa

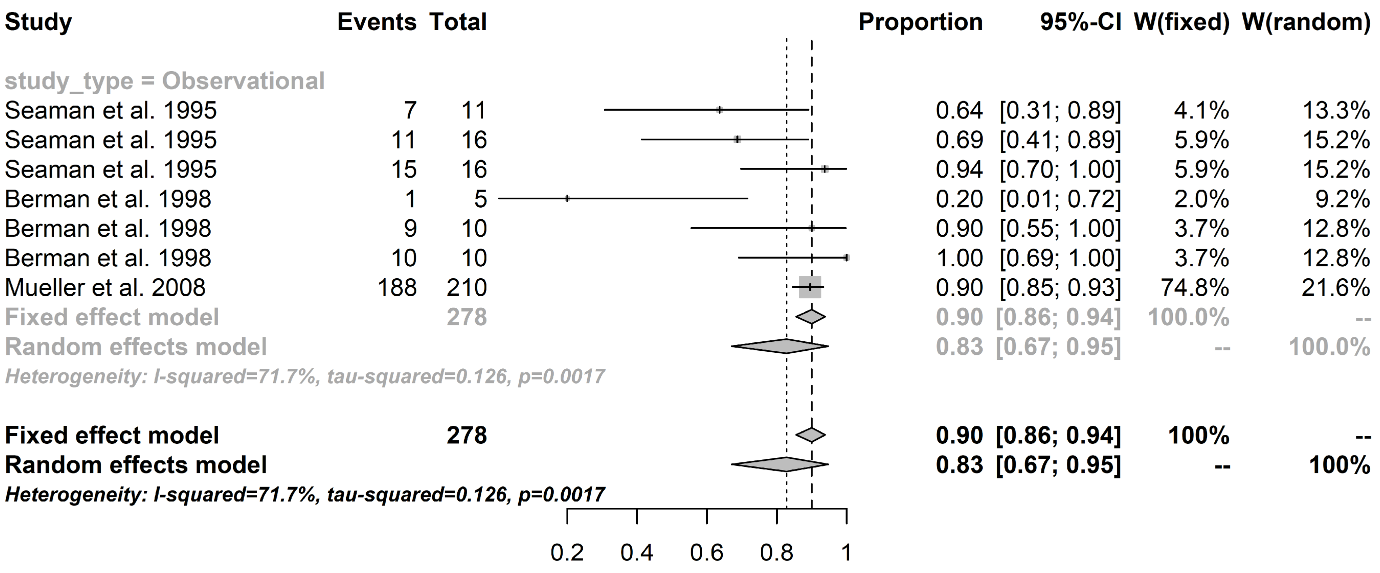

5. Forest plot comparing proportions cured within study-arm for treatment with systemic antimony of patients with visceral leishmaniasis caused by *L. donovani* from East Africa

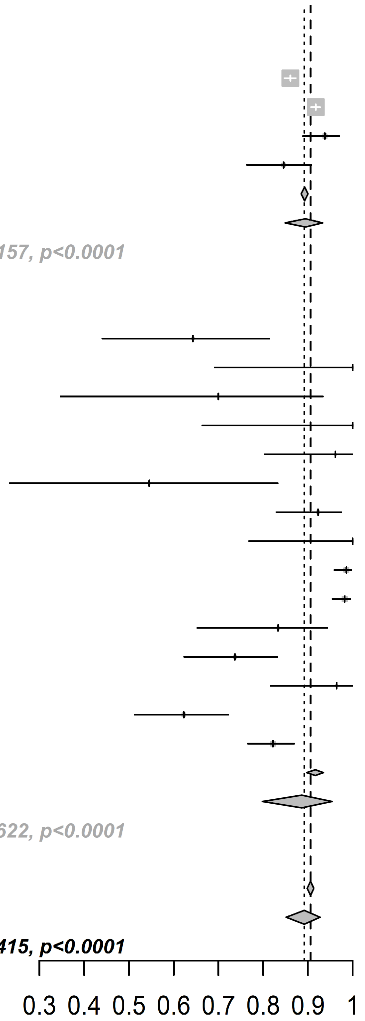

Study	Events	Total	Proportion	95%-CI	W(fixed)	W(random)
study_type = Observational						
Seaman et al. 1996	2562	2977	0.86	[0.85; 0.87]	40.9%	8.0%
Collin et al. 2004	2717	2961	0.92	[0.91; 0.93]	40.7%	8.0%
Mueller et al. 2008	151	161	0.94	[0.89; 0.97]	2.2%	7.0%
Hurissa et al. 2010	93	110	0.85	[0.76; 0.91]	1.5%	6.6%
Fixed effect model		6209	0.89	[0.88; 0.90]	85.3%	--
Random effects model			0.89	[0.85; 0.93]	--	29.6%
Heterogeneity: I-squared=94.6%, tau-squared=0.0157, p<0.0001						
study_type = RCT						
Anabwani et al. 1983	18	28	0.64	[0.44; 0.81]	0.4%	4.4%
Chulay et al. 1983	10	10	1.00	[0.69; 1.00]	0.1%	2.4%
Chulay et al. 1983	7	10	0.70	[0.35; 0.93]	0.1%	2.4%
Chulay et al. 1983	9	9	1.00	[0.66; 1.00]	0.1%	2.3%
Anabwani et al. 1983	25	26	0.96	[0.80; 1.00]	0.4%	4.2%
Chunge et al. 1990	6	11	0.55	[0.23; 0.83]	0.2%	2.6%
Seaman et al. 1993	60	65	0.92	[0.83; 0.97]	0.9%	5.9%
Squires et al. 1993	14	14	1.00	[0.77; 1.00]	0.2%	3.0%
Veeken et al. 2000	210	213	0.99	[0.96; 1.00]	2.9%	7.2%
Veeken et al. 2000	219	223	0.98	[0.95; 1.00]	3.1%	7.3%
Moore et al. 2001	25	30	0.83	[0.65; 0.94]	0.4%	4.5%
Ritmeijer et al. 2001	56	76	0.74	[0.62; 0.83]	1.1%	6.1%
Moore et al. 2001	27	28	0.96	[0.82; 1.00]	0.4%	4.4%
Ritmeijer et al. 2001	56	90	0.62	[0.51; 0.72]	1.2%	6.4%
Ritmeijer et al. 2006	189	230	0.82	[0.77; 0.87]	3.2%	7.3%
Fixed effect model		1063	0.92	[0.90; 0.93]	14.7%	--
Random effects model			0.89	[0.80; 0.95]	--	70.4%
Heterogeneity: I-squared=91.3%, tau-squared=0.1622, p<0.0001						
**Fixed effect model**		**7272**	**0.91**	**[0.90; 0.91]**	**100%**	**--**
**Random effects model**			**0.89**	**[0.85; 0.93]**	**--**	**100%**
Heterogeneity: I-squared=91.8%, tau-squared=0.0415, p<0.0001						

6. Forest plot comparing proportions cured within study-arm for treatment with amphotericin B formulations of pediatric patients with visceral leishmaniasis caused by *L. infantum* from the Old World

Study	Events	Total	Proportion	95%-CI	W(fixed)	W(random)
**study_type = Observational**						
Davidson et al. 1994	6	6	1.00	[0.54; 1.00]	2.4%	2.7%
Davidson et al. 1994	9	9	1.00	[0.66; 1.00]	3.5%	3.9%
Davidson et al. 1996	16	19	0.84	[0.60; 0.97]	7.2%	7.7%
Davidson et al. 1996	25	26	0.96	[0.80; 1.00]	9.9%	10.1%
Davidson et al. 1996	7	7	1.00	[0.59; 1.00]	2.8%	3.1%
Syriopoulou et al. 2003	40	41	0.98	[0.87; 1.00]	15.4%	14.8%
Syriopoulou et al. 2003	27	30	0.90	[0.73; 0.98]	11.3%	11.4%
Harms et al. 2003	6	6	1.00	[0.54; 1.00]	2.4%	2.7%
Kafetzis et al. 2005	6	6	1.00	[0.54; 1.00]	2.4%	2.7%
Kafetzis et al. 2005	6	6	1.00	[0.54; 1.00]	2.4%	2.7%
**Fixed effect model**		**156**	**0.97**	**[0.93; 1.00]**	**59.9%**	**--**
**Random effects model**			**0.97**	**[0.93; 1.00]**	**--**	**61.9%**
Heterogeneity: I-squared=0%, tau-squared=0, p=0.8327						
**study_type = RCT**						
Di Martino et al. 1997	12	16	0.75	[0.48; 0.93]	6.1%	6.6%
Di Martino et al. 1997	65	66	0.98	[0.92; 1.00]	24.7%	21.3%
Di Martino et al. 1997	11	11	1.00	[0.72; 1.00]	4.3%	4.7%
Di Martino et al. 1997	13	13	1.00	[0.75; 1.00]	5.0%	5.5%
**Fixed effect model**		**106**	**0.98**	**[0.94; 1.00]**	**40.1%**	**--**
**Random effects model**			**0.97**	**[0.85; 1.00]**	**--**	**38.1%**
Heterogeneity: I-squared=65.4%, tau-squared=0.0922, p=0.0338						
**Fixed effect model**		**262**	**0.98**	**[0.95; 1.00]**	**100%**	**--**
**Random effects model**			**0.98**	**[0.94; 1.00]**	**--**	**100%**
Heterogeneity: I-squared=8.9%, tau-squared=0.0054, p=0.3551						

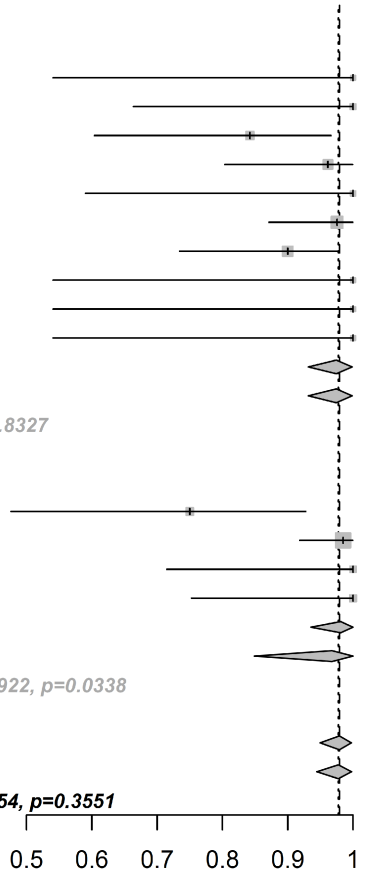

7. Forest plot comparing proportions cured within study-arm for treatment with systemic antimony of pediatric patients with visceral leishmaniasis caused by *L. infantum* from the Old World

Study	Events	Total	Proportion	95%-CI	W(fixed)	W(random)
study_type = Observational						
Syriopoulou et al. 2003	47	52	0.90	[0.79; 0.97]	83.3%	81.3%
Kafetzis et al. 2005	10	10	1.00	[0.69; 1.00]	16.7%	18.7%
Fixed effect model		62	0.94	[0.85; 0.99]	100.0%	--
Random effects model			0.94	[0.85; 0.99]	--	100.0%
Heterogeneity: I-squared=6.2%, tau-squared=0.0038, p=0.3018						
**Fixed effect model**		**62**	**0.94**	**[0.85; 0.99]**	**100%**	**--**
**Random effects model**			**0.94**	**[0.85; 0.99]**	**--**	**100%**
Heterogeneity: I-squared=6.2%, tau-squared=0.0038, p=0.3018						

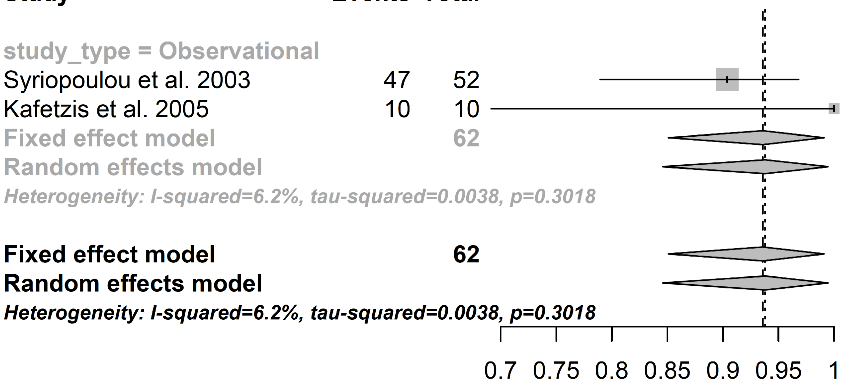

8. Forest plot comparing proportions cured within study-arm for treatment with amphotericin B formulations of adult patients with visceral leishmaniasis caused by *L. infantum* from the Old World

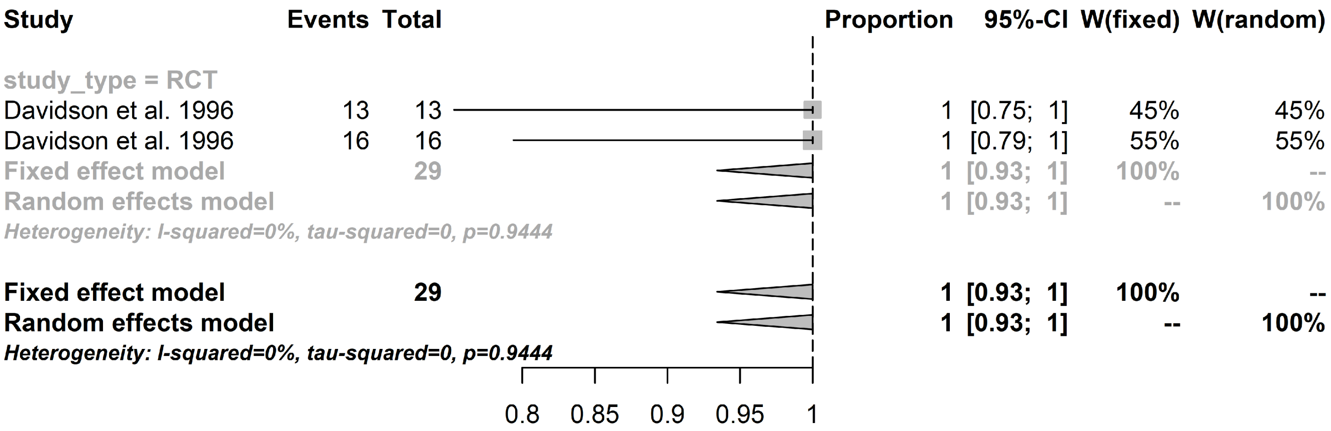

9. Forest plot comparing proportions cured within study-arm for treatment with intralesional antimony of patients with cutaneous leishmaniasis caused by *L. tropica*

Study	Events	Total	Proportion	95%-CI	W(fixed)	W(random)
study_type = Observational						
Ghosn et al. 1979	11	16	0.69	[0.41; 0.89]	1.9%	16.6%
Uzun et al. 2004	504	540	0.93	[0.91; 0.95]	61.0%	21.8%
Fixed effect model		556	0.94	[0.92; 0.96]	62.9%	--
Random effects model			0.85	[0.54; 1.00]	--	38.5%
Heterogeneity: I-squared=86.6%, tau-squared=0.2026, p=0.0062						
study_type = RCT						
Reithinger et al. 2005	70	93	0.75	[0.65; 0.84]	10.6%	20.8%
Layegh et al. 2009	10	36	0.28	[0.14; 0.45]	4.1%	19.2%
Bumb et al. 2010	182	198	0.92	[0.87; 0.95]	22.4%	21.5%
Fixed effect model		327	0.83	[0.78; 0.87]	37.1%	--
Random effects model			0.68	[0.34; 0.94]	--	61.5%
Heterogeneity: I-squared=97%, tau-squared=0.3669, p<0.0001						
**Fixed effect model**		**883**	**0.90**	**[0.88; 0.92]**	**100%**	**--**
**Random effects model**			**0.76**	**[0.56; 0.91]**	**--**	**100%**
Heterogeneity: I-squared=95.9%, tau-squared=0.1866, p<0.0001						

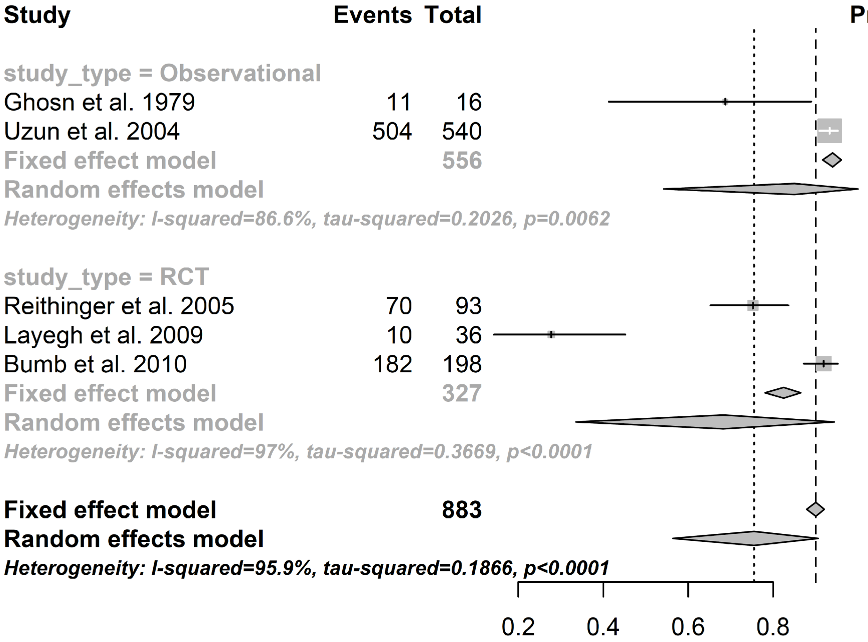

10. Forest plot comparing proportions cured within study-arm for treatment with systemic antimony of patients with cutaneous leishmaniasis caused by *L. tropica*

Study	Events	Total	Proportion	95%-CI	W(fixed)	W(random)
**study_type = Observational**						
Ghosn et al. 1979	8	10	0.80	[0.44; 0.97]	3.9%	10.8%
Layegh et al. 2011	49	100	0.49	[0.39; 0.59]	37.3%	24.5%
Fixed effect model		110	0.52	[0.42; 0.62]	41.2%	--
Random effects model			0.61	[0.31; 0.87]	--	35.3%
Heterogeneity: I-squared=70.8%, tau-squared=0.1278, p=0.064						
**study_type = RCT**						
Esfandiarpour et al. 2002	12	50	0.24	[0.13; 0.38]	18.7%	21.4%
Reithinger et al. 2005	26	58	0.45	[0.32; 0.58]	21.7%	22.1%
Firooz et al. 2006	24	49	0.49	[0.34; 0.64]	18.4%	21.2%
Fixed effect model		157	0.39	[0.32; 0.47]	58.8%	--
Random effects model			0.39	[0.24; 0.55]	--	64.7%
Heterogeneity: I-squared=74.4%, tau-squared=0.0551, p=0.0202						
**Fixed effect model**		**267**	**0.44**	**[0.38; 0.50]**	**100%**	**--**
**Random effects model**			**0.46**	**[0.33; 0.59]**	**--**	**100%**
Heterogeneity: I-squared=74%, tau-squared=0.0567, p=0.004						

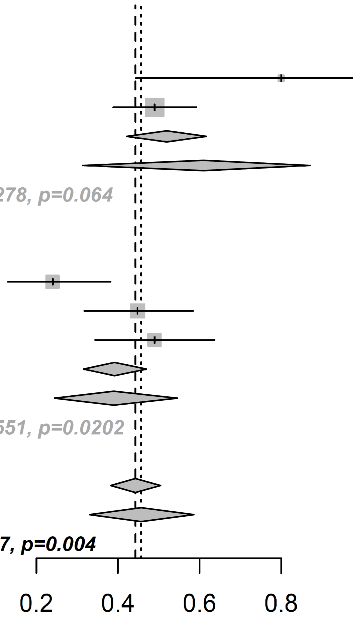

11. Forest plot comparing proportions cured within study-arm for treatment with intralesional antimony of patients with cutaneous leishmaniasis caused by *L. major*

Study	Events	Total	Proportion	95%-CI	W(fixed)	W(random)
study_type = Observational						
Solomon et al. 2009	30	33	0.91	[0.76; 0.98]	13.2%	24.4%
Van Thiel et al. 2010	65	89	0.73	[0.63; 0.82]	35.2%	25.7%
Fixed effect model		122	0.79	[0.71; 0.86]	48.4%	--
Random effects model			0.82	[0.62; 0.96]	--	50.0%
Heterogeneity: I-squared=79.4%, tau-squared=0.079, p=0.0276						
study_type = RCT						
Shazad et al. 2005	18	30	0.60	[0.41; 0.77]	12.0%	24.2%
Moosavi et al. 2005 Int J Derm	100	100	1.00	[0.96; 1.00]	39.6%	25.7%
Fixed effect model		130	0.97	[0.93; 1.00]	51.6%	--
Random effects model			0.88	[0.30; 1.00]	--	50.0%
Heterogeneity: I-squared=97.4%, tau-squared=0.793, p<0.0001						
**Fixed effect model**		**252**	**0.90**	**[0.86; 0.93]**	**100%**	**--**
**Random effects model**			**0.86**	**[0.59; 1.00]**	**--**	**100%**
Heterogeneity: I-squared=95.3%, tau-squared=0.3508, p<0.0001						

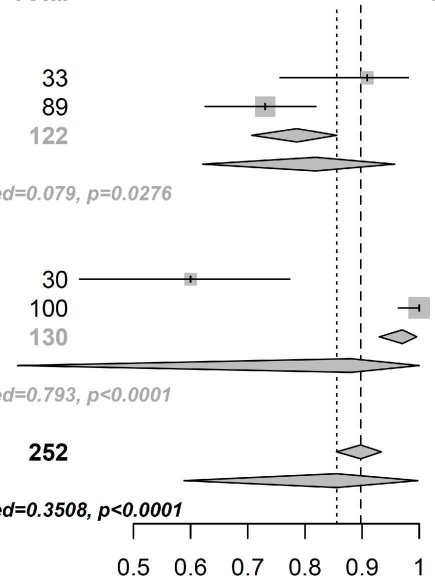

12. Forest plot comparing proportions cured within study-arm for treatment with miltefosine of patients with cutaneous leishmaniasis caused by *L. major*

Study	Events	Total	Proportion	95%-CI	W(fixed)	W(random)
study_type = Observational						
Van Thiel et al. 2010	30	34	0.88	[0.73; 0.97]	51.5%	51.5%
Fixed effect model		34	0.88	[0.75; 0.97]	51.5%	--
Random effects model			0.88	[0.75; 0.97]	--	51.5%
Heterogeneity: I-squared=NaN%, tau-squared=0, p=1						
study_type = RCT						
Mohebali et al. 2007	26	32	0.81	[0.64; 0.93]	48.5%	48.5%
Fixed effect model		32	0.81	[0.66; 0.93]	48.5%	--
Random effects model			0.81	[0.66; 0.93]	--	48.5%
Heterogeneity: I-squared=NaN%, tau-squared=0, p=1						
**Fixed effect model**		**66**	**0.85**	**[0.75; 0.93]**	**100%**	**--**
**Random effects model**			**0.85**	**[0.75; 0.93]**	**--**	**100%**
Heterogeneity: I-squared=0%, tau-squared=0, p=0.445						

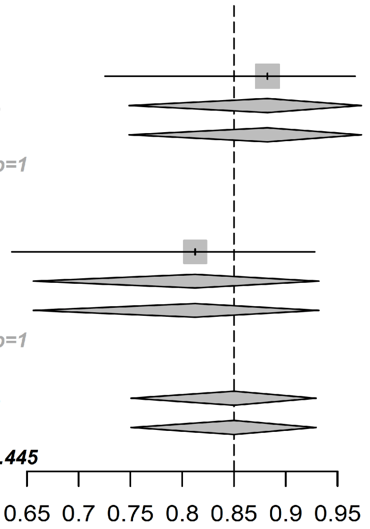

13. Forest plot comparing proportions cured within study-arm for treatment with systemic antimony of patients with cutaneous leishmaniasis caused by *L. major*

Study	Events	Total	Proportion	95%-CI	W(fixed)	W(random)
**study_type = Observational**						
Firdous et al. 2009	182	207	0.88	[0.83; 0.92]	46.1%	15.8%
Solomon et al. 2009	18	21	0.86	[0.64; 0.97]	4.8%	13.1%
**Fixed effect model**		**228**	**0.88**	**[0.84; 0.92]**	**50.8%**	--
**Random effects model**			**0.88**	**[0.84; 0.92]**	--	**28.9%**
Heterogeneity: I-squared=0%, tau-squared=0, p=0.6483						
**study_type = RCT**						
Belazzoug et al. 1986	47	97	0.48	[0.38; 0.59]	21.6%	15.4%
Momeni et al. 2002	21	35	0.60	[0.42; 0.76]	7.9%	14.2%
Sadeghian et al. 2006	16	31	0.52	[0.33; 0.70]	7.0%	14.0%
Mohebali et al. 2007	25	30	0.83	[0.65; 0.94]	6.8%	13.9%
Aronson et al. 2010	14	26	0.54	[0.33; 0.73]	5.9%	13.6%
**Fixed effect model**		**219**	**0.57**	**[0.50; 0.63]**	**49.2%**	--
**Random effects model**			**0.59**	**[0.46; 0.72]**	--	**71.1%**
Heterogeneity: I-squared=69.3%, tau-squared=0.0561, p=0.0111						
**Fixed effect model**		**447**	**0.74**	**[0.70; 0.78]**	**100%**	**--**
**Random effects model**			**0.69**	**[0.51; 0.84]**	**--**	**100%**
Heterogeneity: I-squared=91.6%, tau-squared=0.2013, p<0.0001						

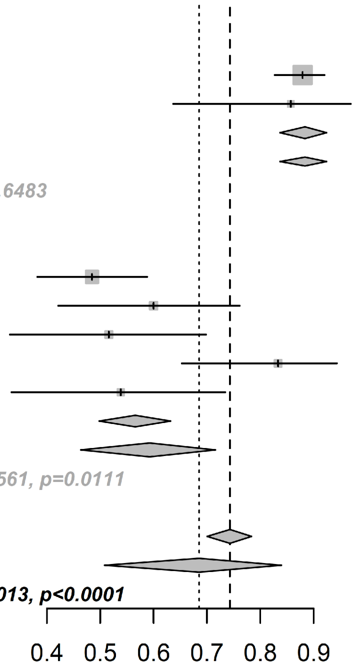

14. Forest plot comparing proportions cured within study-arm for treatment with the combination of intralesional antimony and cryotherapy of patients with cutaneous leishmaniasis caused by *L. major* or *L. tropica*

Study	Events	Total	Proportion	95%-CI	W(fixed)	W(random)
study_type = RCT						
Asilian et al. 2003	60	65	0.92	[0.83; 0.97]	24.7%	24.7%
Asilian et al. 2003	60	67	0.90	[0.80; 0.96]	25.4%	25.4%
Asilian et al. 2004	120	132	0.91	[0.85; 0.95]	49.9%	49.9%
Fixed effect model		264	0.91	[0.87; 0.94]	100.0%	--
Random effects model			0.91	[0.87; 0.94]	--	100.0%
Heterogeneity: I-squared=0%, tau-squared=0, p=0.8677						
**Fixed effect model**		**264**	**0.91**	**[0.87; 0.94]**	**100%**	**--**
**Random effects model**			**0.91**	**[0.87; 0.94]**	**--**	**100%**
Heterogeneity: I-squared=0%, tau-squared=0, p=0.8677						

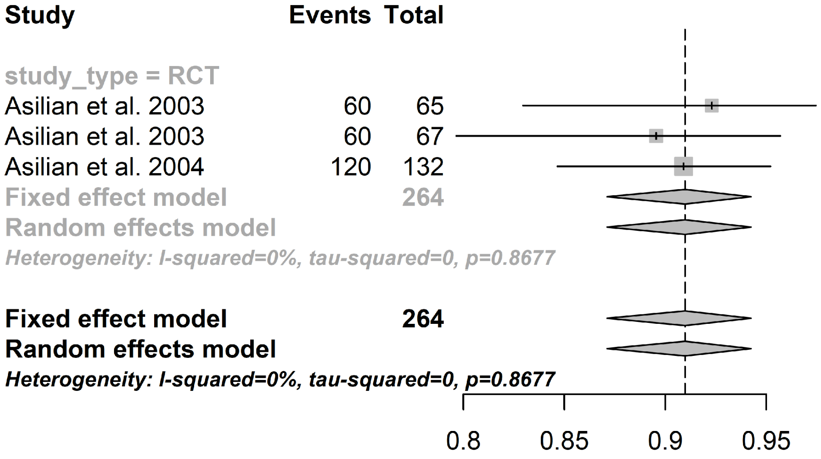

15. Forest plot comparing proportions cured within study-arm for treatment with intralesional antimony of patients with cutaneous leishmaniasis caused by *L. major* or *L. tropica*

Study	Events	Total	Proportion	95%−CI	W(fixed)	W(random)
study_type = RCT						
Salmanpour et al. 2001	23	32	0.72	[0.53; 0.86]	10.6%	13.4%
Faghihi et al. 2003	20	48	0.42	[0.28; 0.57]	15.9%	14.7%
Iraji et al. 2004	21	46	0.46	[0.31; 0.61]	15.2%	14.6%
Nilforoushzadeh et al. 2006	23	35	0.66	[0.48; 0.81]	11.6%	13.7%
Sadeghian et al. 2006	9	36	0.25	[0.12; 0.42]	11.9%	13.8%
Sadeghian et al. 2007	34	60	0.57	[0.43; 0.69]	19.8%	15.3%
Nilforoushzadeh et al. 2007	32	45	0.71	[0.56; 0.84]	14.9%	14.5%
Fixed effect model		302	0.54	[0.48; 0.59]	100.0%	--
Random effects model					--	100.0%
Heterogeneity: I−squared=78.9%, tau−squared=0.0863, p<0.0001						
**Fixed effect model**		**302**	**0.54**	**[0.48; 0.59]**	**100%**	**--**
**Random effects model**			**0.54**	**[0.41; 0.66]**	**--**	**100%**
Heterogeneity: I−squared=78.9%, tau−squared=0.0863, p<0.0001						

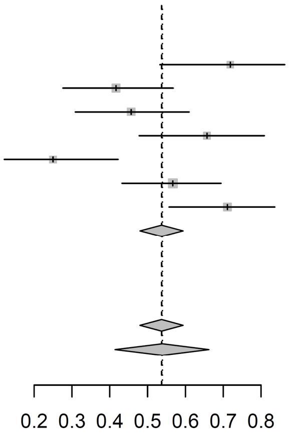

16. Forest plot comparing proportions **of lesions** cured within study-arm for treatment with intralesional antimony of patients with cutaneous leishmaniasis caused by *L. major* or *L. tropica*

Study	Events	Total	Proportion	95%-CI	W(fixed)	W(random)
study_type = Observational						
Sharquie et al. 1988	123	130	0.95	[0.89; 0.98]	7.1%	11.6%
Fixed effect model		130	0.95	[0.90; 0.98]	7.1%	--
Random effects model			0.95	[0.90; 0.98]	--	11.6%
Heterogeneity: I-squared=NaN%, tau-squared=0, p=1						
study_type = RCT						
Faris et al. 1993	756	1050	0.72	[0.69; 0.75]	57.4%	12.1%
Sharquie et al. 1995	27	28	0.96	[0.82; 1.00]	1.6%	9.7%
Sharquie et al. 1997	31	35	0.89	[0.73; 0.97]	1.9%	10.1%
Sharquie et al. 1998	32	36	0.89	[0.74; 0.97]	2.0%	10.2%
Mujtaba et al. 1999	102	111	0.92	[0.85; 0.96]	6.1%	11.5%
Mujtaba et al. 1999	89	104	0.86	[0.77; 0.92]	5.7%	11.4%
Asilian et al. 2003	90	180	0.50	[0.42; 0.58]	9.9%	11.7%
Asilian et al. 2004	84	151	0.56	[0.47; 0.64]	8.3%	11.7%
Fixed effect model		1695	0.72	[0.70; 0.75]	92.9%	--
Random effects model			0.80	[0.68; 0.89]	--	88.4%
Heterogeneity: I-squared=94.2%, tau-squared=0.114, p<0.0001						
**Fixed effect model**		**1825**	**0.74**	**[0.72; 0.76]**	**100%**	**--**
**Random effects model**			**0.82**	**[0.71; 0.91]**	**--**	**100%**
Heterogeneity: I-squared=95.3%, tau-squared=0.1374, p<0.0001						

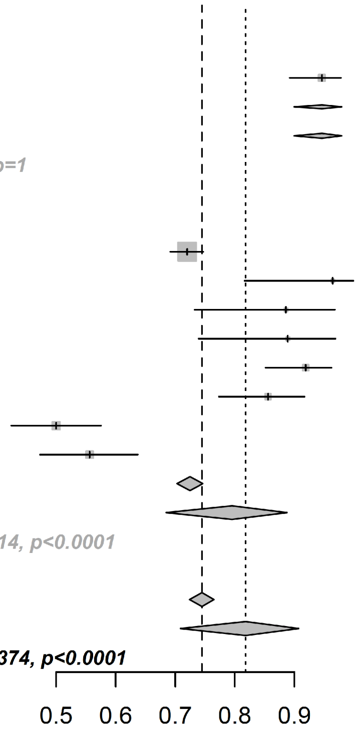

17. Forest plot comparing proportions cured within study-arm for treatment with heat therapy of patients with cutaneous leishmaniasis caused by *L. major* or *L. tropica*

Study	Events	Total	Proportion	95%-CI	W(fixed)	W(random)
study_type = Observational						
Aram et al. 1987	13	18	0.72	[0.47; 0.90]	19.0%	19.3%
Asilian et al. 2006	19	21	0.90	[0.70; 0.99]	22.1%	22.4%
Fixed effect model		39	0.83	[0.69; 0.94]	41.0%	--
Random effects model			0.83	[0.62; 0.97]	--	41.7%
Heterogeneity: I-squared=50.5%, tau-squared=0.0514, p=0.1551						
study_type = RCT						
Sadeghian et al. 2007	46	57	0.81	[0.68; 0.90]	59.0%	58.3%
Fixed effect model		57	0.81	[0.69; 0.90]	59.0%	--
Random effects model			0.81	[0.69; 0.90]	--	58.3%
Heterogeneity: I-squared=NaN%, tau-squared=0, p=1						
**Fixed effect model**		**96**	**0.82**	**[0.73; 0.89]**	**100%**	**--**
**Random effects model**			**0.82**	**[0.73; 0.89]**	**--**	**100%**
Heterogeneity: I-squared=2.1%, tau-squared=0.0008, p=0.3601						

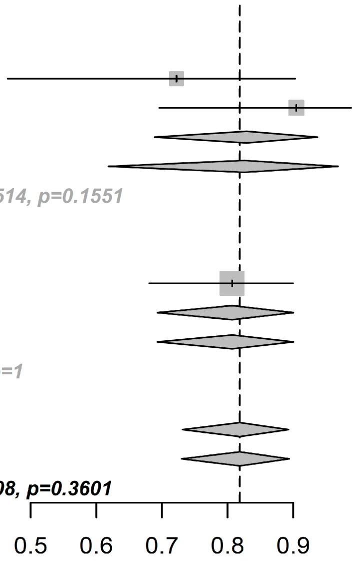

18. Forest plot comparing proportions **of lesions** cured within study-arm for treatment with heat therapy of patients with cutaneous leishmaniasis caused by *L. major* or *L. tropica*

Study	Events	Total	Proportion	95%-CI	W(fixed)	W(random)
study_type = RCT						
Sharquie et al. 1998	135	146	0.92	[0.87; 0.96]	56.8%	56.8%
Asilian et al. 2004	104	111	0.94	[0.87; 0.97]	43.2%	43.2%
Fixed effect model		257	0.93	[0.89; 0.96]	100.0%	--
Random effects model			0.93	[0.89; 0.96]	--	100.0%
Heterogeneity: I-squared=0%, tau-squared=0, p=0.7286						
**Fixed effect model**		**257**	**0.93**	**[0.89; 0.96]**	**100%**	**--**
**Random effects model**			**0.93**	**[0.89; 0.96]**	**--**	**100%**
Heterogeneity: I-squared=0%, tau-squared=0, p=0.7286						

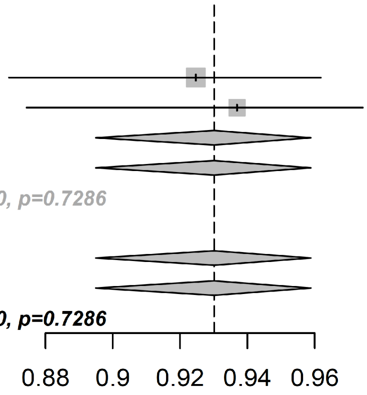

19. Forest plot comparing proportions cured within study-arm for treatment with systemic antimony of patients with cutaneous leishmaniasis caused by *L. major* or *L. tropica*

Study	Events	Total	Proportion	95%-CI	W(fixed)	W(random)
study_type = RCT						
Zerehsaz et al. 1999	23	85	0.27	[0.18; 0.38]	66.3%	50.3%
Nilforoushzadeh et al. 2008	40	43	0.93	[0.81; 0.99]	33.7%	49.7%
Fixed effect model		128	0.51	[0.43; 0.60]	100.0%	--
Random effects model			0.63	[0.03; 1.00]	--	100.0%
Heterogeneity: I-squared=98.4%, tau-squared=1.065, p<0.0001						
**Fixed effect model**		**128**	**0.51**	**[0.43; 0.60]**	**100%**	**--**
**Random effects model**			**0.63**	**[0.03; 1.00]**	**--**	**100%**
Heterogeneity: I-squared=98.4%, tau-squared=1.065, p<0.0001						

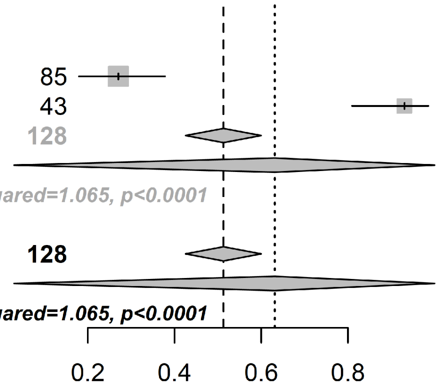

20. Forest plot comparing proportions **of lesions** cured within study-arm for treatment with systemic antimony of patients with cutaneous leishmaniasis caused by *L. major* or *L. tropica*

Study	Events	Total	Proportion	95%-CI	W(fixed)	W(random)
study_type = RCT						
Asilian et al. 2004	176	210	0.84	[0.78; 0.89]	78.3%	50.6%
Layegh et al. 2007	20	58	0.34	[0.22; 0.48]	21.7%	49.4%
Fixed effect model		268	0.75	[0.69; 0.80]	100.0%	--
Random effects model			0.61	[0.13; 0.98]	--	100.0%
Heterogeneity: I-squared=98%, tau-squared=0.5386, p<0.0001						
**Fixed effect model**		**268**	**0.75**	**[0.69; 0.80]**	**100%**	**--**
**Random effects model**			**0.61**	**[0.13; 0.98]**	**--**	**100%**
Heterogeneity: I-squared=98%, tau-squared=0.5386, p<0.0001						

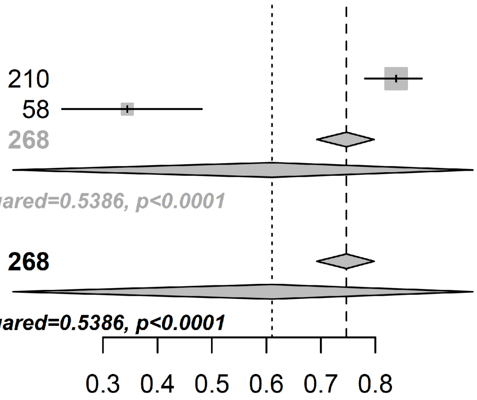

21. Forest plot comparing proportions cured within study-arm for treatment with amphotericin B formulations of patients with visceral leishmaniasis caused by *L. infantum* from South America (“*L. chagasi*”)

Study	Events	Total	Proportion	95%-CI	W(fixed)	W(random)
study_type = Observational						
Dietze et al. 1993	10	10	1	[0.69; 1]	32.3%	32.3%
Dietze et al. 1993	10	10	1	[0.69; 1]	32.3%	32.3%
Dietze et al. 1995	11	11	1	[0.72; 1]	35.4%	35.4%
Fixed effect model		31	1	[0.93; 1]	100.0%	--
Random effects model			1	[0.93; 1]	--	100.0%
Heterogeneity: I-squared=0%, tau-squared=0, p=0.9993						
**Fixed effect model**		**31**	**1**	**[0.93; 1]**	**100%**	**--**
**Random effects model**			**1**	**[0.93; 1]**	**--**	**100%**
Heterogeneity: I-squared=0%, tau-squared=0, p=0.9993						

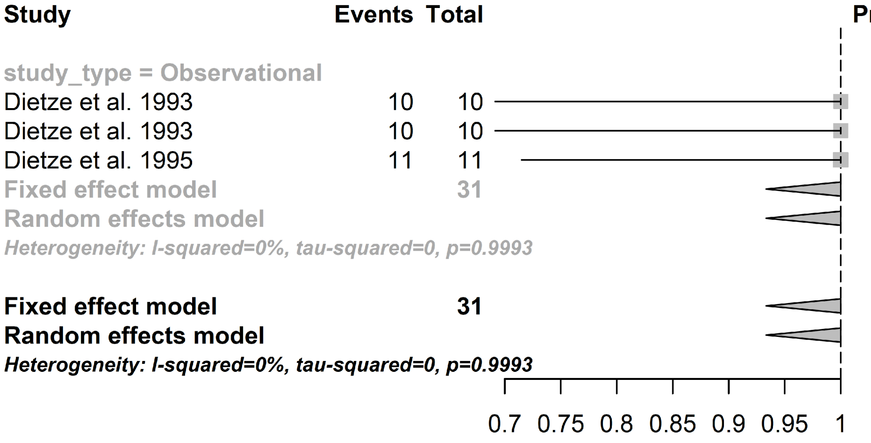

22. Forest plot comparing proportions cured within study-arm for treatment with systemic antimony of patients with cutaneous leishmaniasis caused by *L. mexicana*

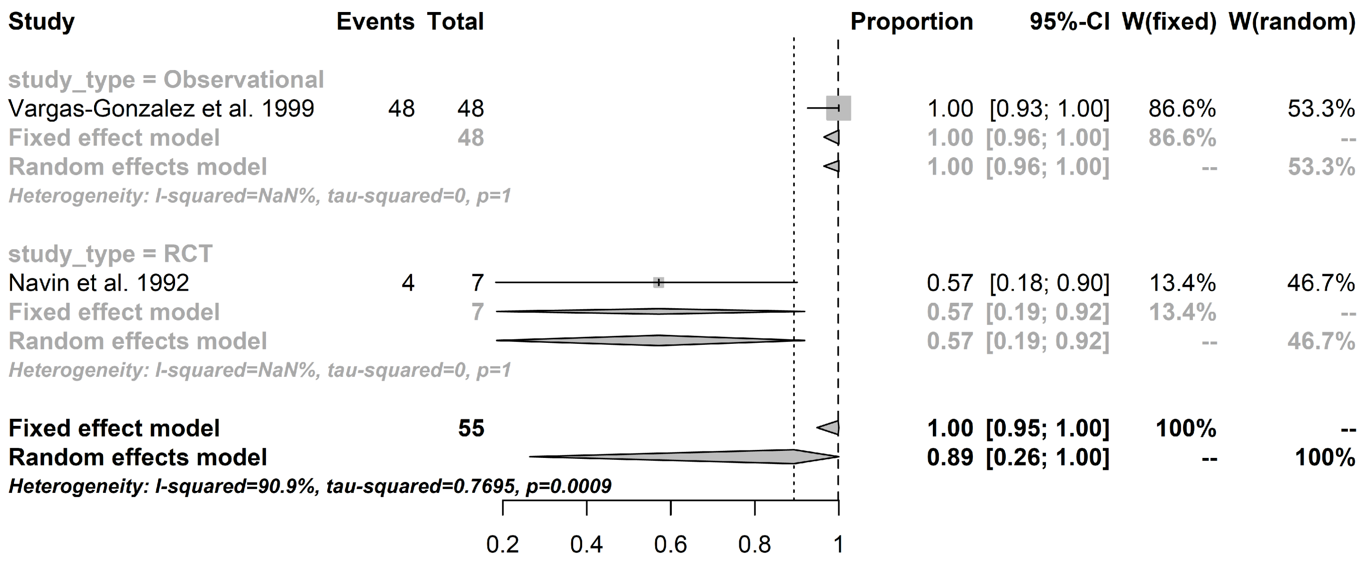

23. Forest plot comparing proportions cured within study-arm for treatment with systemic antimony of patients with cutaneous leishmaniasis caused by *L. braziliensis*

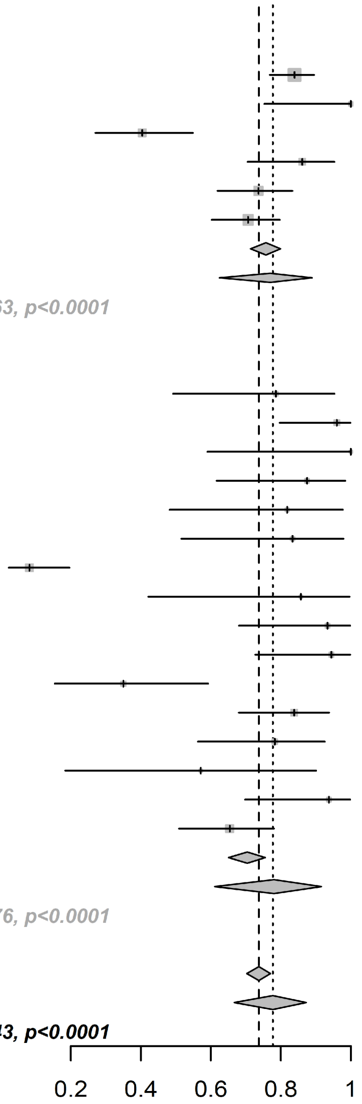

Study	Events	Total	Proportion	95%-CI	W(fixed)	W(random)
**study_type = Observational**						
Oliveira-Neto et al. 1997	120	143	0.84	[0.77; 0.90]	19.2%	5.3%
Seaton et al. 1999	13	13	1.00	[0.75; 1.00]	1.8%	4.2%
Romero et al. 2001	21	52	0.40	[0.27; 0.55]	7.0%	5.1%
Oliveira-Neto et al. 2006	31	36	0.86	[0.71; 0.95]	4.9%	4.9%
Arevalo et al. 2007	53	72	0.74	[0.62; 0.83]	9.7%	5.2%
Llanos-Cuentas et al. 2008	65	92	0.71	[0.60; 0.80]	12.4%	5.2%
**Fixed effect model**		**408**	**0.76**	**[0.71; 0.80]**	**54.9%**	**--**
**Random effects model**			**0.77**	**[0.62; 0.89]**	**--**	**30.0%**
Heterogeneity: I-squared=88.9%, tau-squared=0.1263, p<0.0001						
**study_type = RCT**						
Navin et al. 1990	11	14	0.79	[0.49; 0.95]	1.9%	4.3%
Navin et al. 1992	24	25	0.96	[0.80; 1.00]	3.4%	4.7%
Hepburn et al. 1994	7	7	1.00	[0.59; 1.00]	1.0%	3.6%
Correia et al. 1996	14	16	0.88	[0.62; 0.98]	2.2%	4.4%
Oliveira-Neto et al. 1997	9	11	0.82	[0.48; 0.98]	1.5%	4.1%
Oliveira-Neto et al. 1997	10	12	0.83	[0.52; 0.98]	1.7%	4.2%
Machado-Pinto et al. 2002	4	49	0.08	[0.02; 0.20]	6.6%	5.1%
Soto et al. 2004	6	7	0.86	[0.42; 1.00]	1.0%	3.6%
Soto et al. 2004	14	15	0.93	[0.68; 1.00]	2.1%	4.4%
Soto et al. 2004	17	18	0.94	[0.73; 1.00]	2.5%	4.5%
Miranda-Verástegui et al. 2005	7	20	0.35	[0.15; 0.59]	2.7%	4.6%
Andersen et al. 2005	31	37	0.84	[0.68; 0.94]	5.0%	4.9%
Krolewiecki et al. 2007	18	23	0.78	[0.56; 0.93]	3.1%	4.7%
Arevalo et al. 2007	4	7	0.57	[0.18; 0.90]	1.0%	3.6%
Soto et al. 2008	15	16	0.94	[0.70; 1.00]	2.2%	4.4%
Velez et al. 2010	34	52	0.65	[0.51; 0.78]	7.0%	5.1%
**Fixed effect model**		**329**	**0.70**	**[0.65; 0.76]**	**45.1%**	**--**
**Random effects model**			**0.78**	**[0.61; 0.92]**	**--**	**70.0%**
Heterogeneity: I-squared=89.8%, tau-squared=0.4276, p<0.0001						
**Fixed effect model**		**737**	**0.74**	**[0.70; 0.77]**	**100%**	**--**
**Random effects model**			**0.78**	**[0.67; 0.87]**	**--**	**100%**
Heterogeneity: I-squared=89.2%, tau-squared=0.2543, p<0.0001						

24. Forest plot comparing proportions cured within study-arm for treatment with miltefosine of patients with cutaneous leishmaniasis caused by *L. braziliensis*

Study	Events	Total	Proportion	95%-CI	W(fixed)	W(random)
study_type = RCT						
Soto et al. 2004	5	15	0.33	[0.12; 0.62]	14.0%	31.1%
Soto et al. 2008	39	43	0.91	[0.78; 0.97]	39.4%	34.3%
Velez et al. 2010	25	51	0.49	[0.35; 0.63]	46.6%	34.6%
Fixed effect model		109	0.66	[0.56; 0.75]	100.0%	--
Random effects model			0.61	[0.24; 0.92]	--	100.0%
Heterogeneity: I-squared=92.8%, tau-squared=0.3858, p<0.0001						
**Fixed effect model**		**109**	**0.66**	**[0.56; 0.75]**	**100%**	**--**
**Random effects model**			**0.61**	**[0.24; 0.92]**	**--**	**100%**
Heterogeneity: I-squared=92.8%, tau-squared=0.3858, p<0.0001						

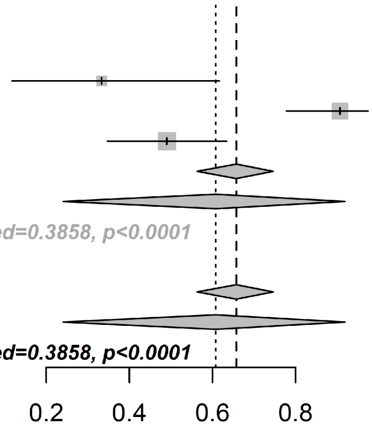

25. Forest plot comparing proportions cured within study-arm for treatment with the combination of systemic antimony and pentoxifylline of patients with mucocutaneous leishmaniasis caused by *L. braziliensis*

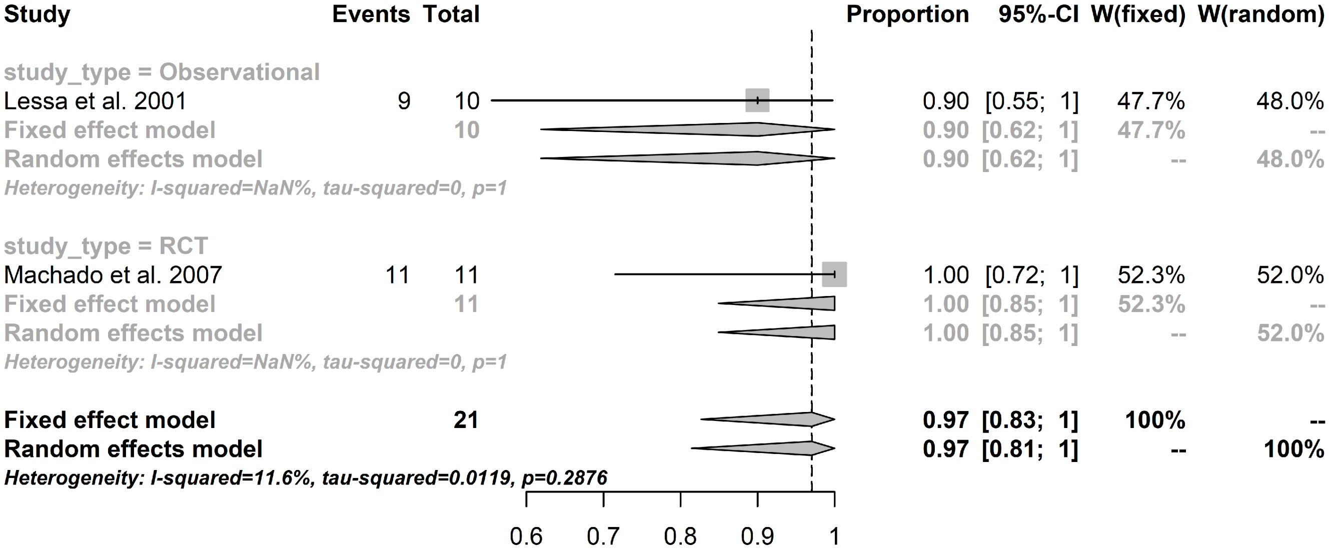

26. Forest plot comparing proportions cured within study-arm for treatment with systemic antimony of patients with mucocutaneous leishmaniasis caused by *L. braziliensis*

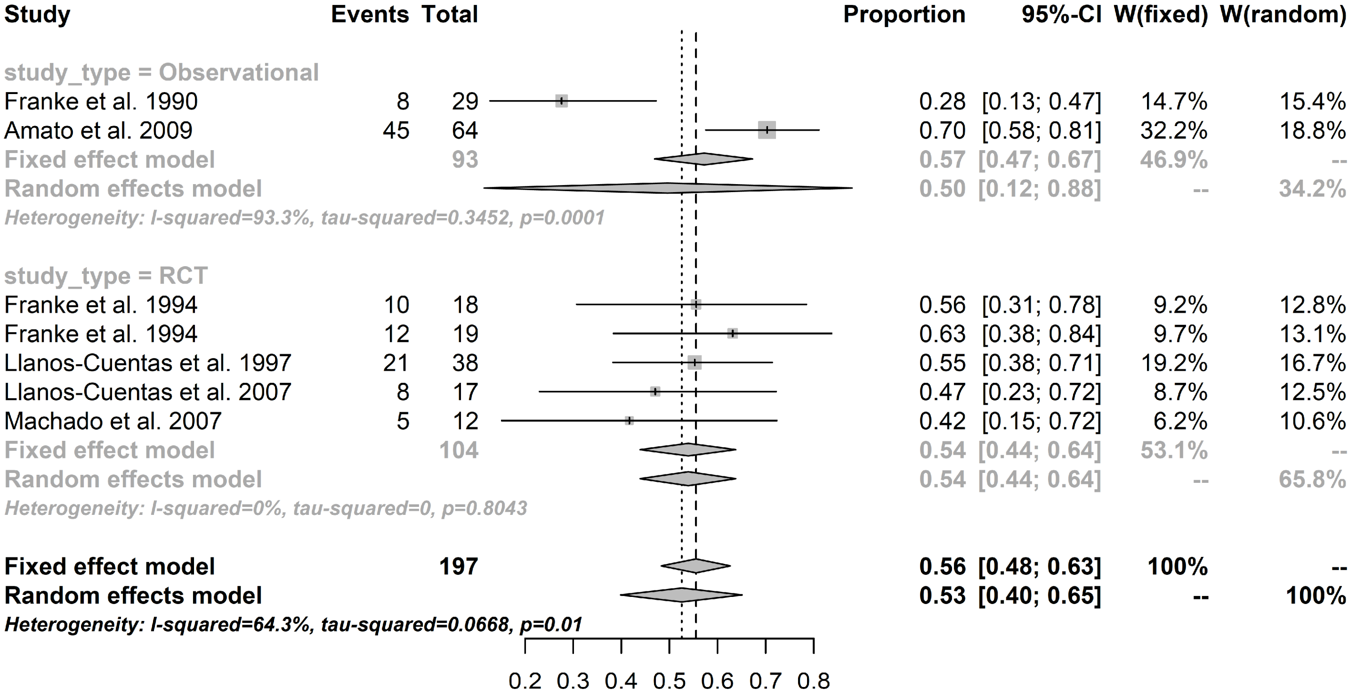

27. Forest plot comparing proportions cured within study-arm for treatment with amphotericin B formulations of patients with mucocutaneous leishmaniasis caused by *L. braziliensis*

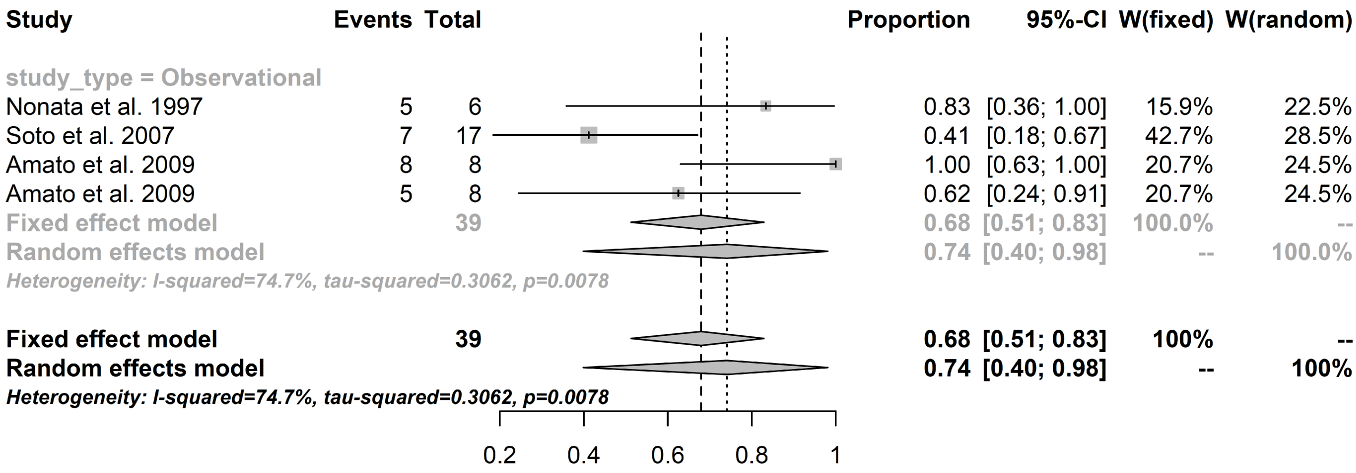

28. Forest plot comparing proportions cured within study-arm for treatment with miltefosine of patients with mucocutaneous leishmaniasis caused by *L. braziliensis*

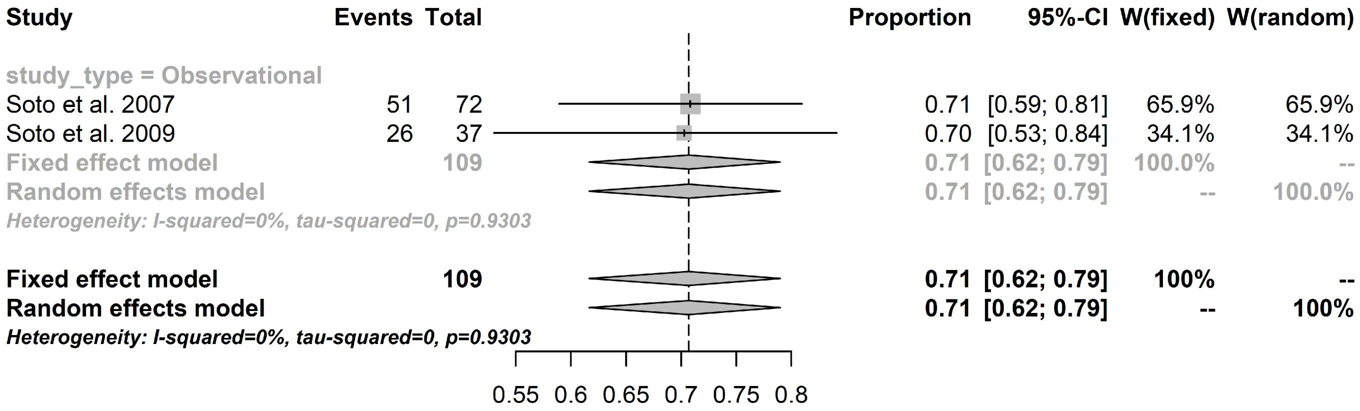

29. Forest plot comparing proportions cured within study-arm for treatment with systemic antimony of patients with cutaneous leishmaniasis caused by *L. panamensis*

Study	Events	Total	Proportion	95%-CI	W(fixed)	W(random)
study_type = Observational						
Hendrickx et al. 1998	44	51	0.86	[0.74; 0.94]	9.0%	8.6%
Soto et al. 2005	184	226	0.81	[0.76; 0.86]	39.4%	9.3%
Fixed effect model		277	0.82	[0.78; 0.87]	48.4%	--
Random effects model			0.82	[0.78; 0.87]	--	17.9%
Heterogeneity: I-squared=0%, tau-squared=0, p=0.4516						
study_type = RCT						
Ballou et al. 1987	16	21	0.76	[0.53; 0.92]	3.7%	7.4%
Ballou et al. 1987	19	19	1.00	[0.82; 1.00]	3.4%	7.3%
Saenz et al. 1990	13	19	0.68	[0.43; 0.87]	3.4%	7.3%
Martinez et al. 1992	12	33	0.36	[0.20; 0.55]	5.8%	8.1%
Martinez et al. 1997	19	48	0.40	[0.26; 0.55]	8.4%	8.5%
Palacios et al. 2001	28	46	0.61	[0.45; 0.75]	8.1%	8.5%
Palacios et al. 2001	24	36	0.67	[0.49; 0.81]	6.4%	8.2%
Wortmann et al. 2002	9	9	1.00	[0.66; 1.00]	1.7%	5.8%
Wortmann et al. 2002	9	9	1.00	[0.66; 1.00]	1.7%	5.8%
Velez et al. 2010	23	32	0.72	[0.53; 0.86]	5.7%	8.0%
Rubiano et al. 2012	12	19	0.63	[0.38; 0.84]	3.4%	7.3%
Fixed effect model		291	0.66	[0.60; 0.71]	51.6%	--
Random effects model			0.73	[0.58; 0.86]	--	82.1%
Heterogeneity: I-squared=84.3%, tau-squared=0.2043, p<0.0001						
**Fixed effect model**		**568**	**0.75**	**[0.71; 0.78]**	**100%**	**--**
**Random effects model**			**0.75**	**[0.63; 0.85]**	**--**	**100%**
Heterogeneity: I-squared=86.1%, tau-squared=0.1606, p<0.0001						

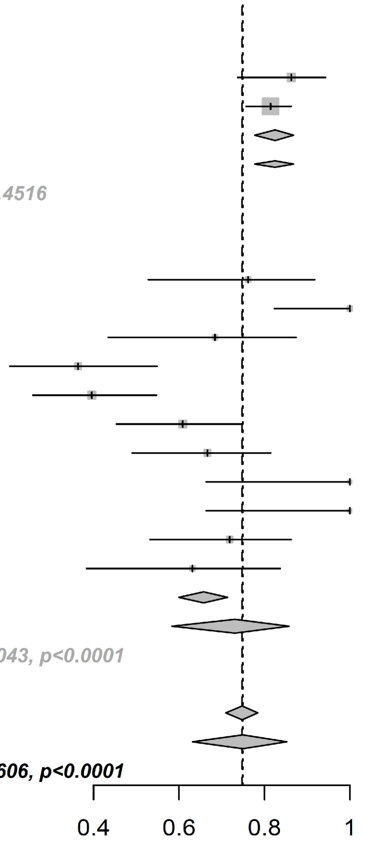

30. Forest plot comparing proportions cured within study-arm for treatment with miltefosine of patients with cutaneous leishmaniasis caused by *L. panamensis*

Study	Events	Total	Proportion	95%-CI	W(fixed)	W(random)
study_type = RCT						
Soto et al. 2004	40	44	0.91	[0.78; 0.97]	44.7%	35.2%
Velez et al. 2010	18	30	0.60	[0.41; 0.77]	30.7%	33.1%
Rubiano et al. 2012	22	24	0.92	[0.73; 0.99]	24.6%	31.7%
Fixed effect model		98	0.83	[0.75; 0.90]	100.0%	--
Random effects model			0.83	[0.61; 0.97]	--	100.0%
Heterogeneity: I-squared=82.5%, tau-squared=0.1463, p=0.0034						
**Fixed effect model**		**98**	**0.83**	**[0.75; 0.90]**	**100%**	**--**
**Random effects model**			**0.83**	**[0.61; 0.97]**	**--**	**100%**
Heterogeneity: I-squared=82.5%, tau-squared=0.1463, p=0.0034						

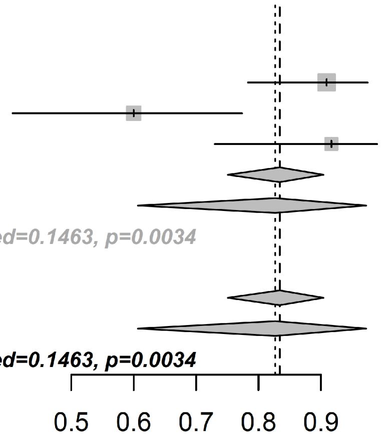

31. Forest plot comparing proportions cured within study-arm for treatment with pentamidine of patients with cutaneous leishmaniasis caused by *L. guyanensis*

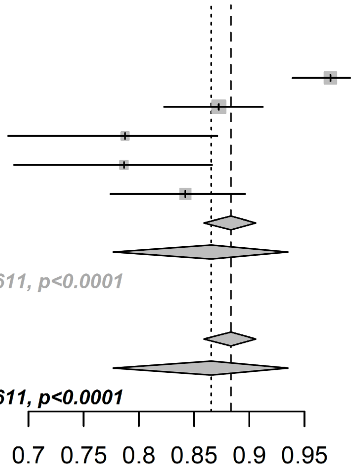

Study	Events	Total	Proportion	95%-CI	W(fixed)	W(random)
study_type = Observational						
Nacher et al. 2001	184	189	0.97	[0.94; 0.99]	25.4%	20.8%
Lai A Fat et al. 2002	205	235	0.87	[0.82; 0.91]	31.5%	21.1%
Lai A Fat et al. 2002	63	80	0.79	[0.68; 0.87]	10.8%	18.7%
Roussel et al. 2006	70	89	0.79	[0.69; 0.87]	12.0%	19.1%
Roussel et al. 2006	128	152	0.84	[0.77; 0.90]	20.4%	20.4%
Fixed effect model		745	0.88	[0.86; 0.91]	100.0%	--
Random effects model			0.87	[0.78; 0.93]	--	100.0%
Heterogeneity: I-squared=89.8%, tau-squared=0.0611, p<0.0001						
**Fixed effect model**		**745**	**0.88**	**[0.86; 0.91]**	**100%**	**--**
**Random effects model**			**0.87**	**[0.78; 0.93]**	**--**	**100%**
Heterogeneity: I-squared=89.8%, tau-squared=0.0611, p<0.0001						
